# Deletion of *FgSRE1* Impairs Reproduction and Virulence of *Fusarium graminearum* and Alters Metabolic and Membrane-Transport Gene Expression

**DOI:** 10.3390/jof12080607

**Published:** 2026-08-14

**Authors:** Linru Shen, Hongyan Hui, Lei Guo, Junshen Wang, Jianchao Cui, Haijiao Xu, Xiaolong He, Yufei Huang, Bo Liu

**Affiliations:** 1 College of Life Sciences, Yan’an University, Yan’an 716000, China; slr13623823842@126.com (L.S.); hhy554999333@126.com (H.H.); m13991239140@163.com (L.G.); 15891783352@163.com (J.W.); ydhelong@163.com (X.H.); 2 Research and Development Centre of Ecological and Sustainable Application of Microbial Industry of the Loess Plateau in Shaanxi Province, Yan’an 716000, China; 3 Changli Institute of Pomology, Hebei Academy of Agricultural and Forestry Sciences, Changli 066600, China; cjc19880320@126.com (J.C.); xuhaijiao1234@sina.cn (H.X.); 4 College of Plant Protection, Shenyang Agricultural University, Shenyang 110866, China

**Keywords:** *Fusarium graminearum*, transcriptome, pathogenicity, maize ear rot, gene function

## Abstract

*Fusarium graminearum,* the major pathogen causing maize ear rot, severely threatens food security. Targeted gene deletion was used to characterize physiological functions of *FgSRE1* in this pathogen. Transcriptomic profiling was conducted on axenic wild-type and Δ*FgSRE1* cultures, plus maize ear tissues colonized by both strains. Nine core DEGs functionally annotated to transmembrane transport, membrane homeostasis and oxidative stress pathways were selected for qRT-PCR validation, including four upregulated genes encoding putative transferases and five downregulated transporter genes. Phenotypic assays revealed that the Δ*FgSRE1* mutant displayed severe defects in conidial and ascospore production, along with drastically weakened pathogenicity on maize ears and leaves versus wild-type and complemented strains. qRT-PCR analysis confirmed consistent expression trends with the RNA-seq data: four putative transferase-encoding genes were up-regulated, whereas five genes related to membrane and redox transport were repressed. Together, the genetic and phenotypic results demonstrate that *FgSRE1* contributes to reproductive development and virulence in *F. graminearum*, whereas the transcriptomic data suggest potential associations with metabolic, redox, and membrane-transport-related pathways. Our findings provide a basis for investigating the *FgSRE1*-associated virulence network and developing eco-friendly maize ear rot control strategies.

## 1. Introduction

*Fusarium graminearum* is a major causal agent of ear rot in maize and wheat [1]. Maize ear rot, a globally prevalent fungal disease caused by *F. graminearum*, causes kernel shrinkage, severe yield losses, and massive accumulation of deoxynivalenol (DON) and zearalenone (ZEA). These mycotoxins degrade grain quality and pose severe threats to human and livestock health [2,3,4,5]. Maize ear rot widely occurs throughout the growing season in major maize-producing regions of China, causing high disease incidence and substantial agricultural losses [5,6]. No specialized pesticides have been officially registered for this disease, and current control relies heavily on chemical agents, which inevitably cause environmental pollution and food safety risks [7]. Furthermore, maize ear rot predominantly emerges at the late maize growth stage, complicating field management. Accordingly, breeding disease-resistant cultivars represents the most fundamental and sustainable control strategy [8].

Mounting evidence reveals that phytopathogenic fungi secrete effectors to disrupt host cellular structure and physiological functions, facilitating colonization while triggering plant immunity [9,10,11,12]. Among these, effectors that interfere with host lipid metabolism and membrane integrity are particularly important. For example, MoErs1 represses the rice immune protease OsRD21, while CgNLP1 blocks nuclear translocation of the necrosis-related transcription factor HbMYB8-Like [13,14]. In *F. graminearum*, the effector protease *FgTPP1* is highly upregulated during wheat spikelet infection, partially localizes to chloroplasts, and attenuates chitin-mediated MAPK signaling and ROS production [15] while the serine carboxypeptidase FgSCP is essential for full virulence and modulates plant immune responses [16]. Other effectors directly target host membrane integrity: Nep1-like proteins (NLPs) act as cytolytic toxins that disrupt the plant plasma membrane and induce tissue necrosis [17]. In addition, pathogens deploy effectors that target plant secretory pathways or directly reprogram host metabolism: the isochorismatase effectors PsIsc1 and VdIsc1 hydrolyze host isochorismate to suppress salicylate-mediated immunity [18]. These findings highlight that a key virulence strategy of fungal effectors is the disruption of host metabolic homeostasis and membrane integrity. Among the diverse effector families, RNase effectors stand out due to their unique ability to directly degrade host RNA and perturb cellular metabolism.

Rnase effectors constitute a unique class of secreted virulence proteins that directly degrade host RNA, thereby disrupting protein synthesis and metabolic flux. In *Setosphaeria turcica*, the Rnase effector SRE1 is markedly upregulated during early maize infection. It enters plant cells via an N-terminal β-hairpin motif and induces host cell death through its intrinsic Rnase activity. Notably, SRE1 exhibits a dual role by both promoting infection and activating plant immunity, and its cell-death-inducing activity is independent of conventional plant receptor kinases BAK1 and SOBIR1 [19]. Similarly, in *F. graminearum*, the secreted Rnase Fg12 also induces cell death in a BAK1/SOBIR1-independent manner and contributes to virulence [20]. These features distinguish Rnase effectors from classical immune-suppressive effectors and suggest their unique mode of action in manipulating host cellular metabolism.

Bioinformatic analysis revealed that *FgSRE1* (FGSG_11190) of *F. graminearum* belongs to the same ribonuclease ribotoxin superfamily, sharing a predicted conserved fungal_Rnase domain and an N-terminal signal peptide, suggesting that it is a candidate Rnase-type effector. Nevertheless, the functions of Rnase effectors in *Fusarium* species remain unexplored, and whether *FgSRE1* disrupts host metabolic processes during maize ear rot infection is unknown. Transcriptome sequencing has been successfully applied to discover and characterize virulence-related effectors in *F. graminearum* [15,21], making it a powerful tool to decipher the host targets and pathways affected by *FgSRE1* [22,23]. Here, we systematically characterize *FgSRE1* through gene knockout, phenotypic assays, pathogenicity tests, and transcriptomic profiling to uncover how this candidate effector contributes to *F. graminearum* virulence, particularly through its effects on host metabolism and defense responses as revealed by transcriptomic analysis.

## 2. Materials and Methods

### 2.1. Strains and Culture Conditions

Wild-type *F. graminearum* (WT) preserved in the Molecular Plant Pathology Laboratory, School of Life Sciences, Yan’an University, was routinely subcultured on potato dextrose agar (PDA) for follow-up assays. The gene deletion mutant Δ*FgSRE1* and its complementary strain Δ*FgSRE1*-C were generated in this work. Carboxymethyl cellulose (CMC) medium was used for strain incubation to quantify conidial production [24], and carrot agar (CA) plates were applied to test ascospore yields.

### 2.2. Construction of ΔFgSRE1 Deletion and Complemented Mutants

Genomic DNA from WT served as the template to amplify the upstream (1638 bp) and downstream (1162 bp) homologous arms and the native promoter fragment of *FgSRE1* [25]. Specific primers used for fragment amplification are listed in Table S1. The hygromycin resistance cassette was amplified, and all target fragments were purified after agarose gel electrophoresis. Subsequently, these fragments were assembled into a single fusion construct via overlap PCR. PCR amplifications were performed using a PCR thermal cycler (K960; Hangzhou Jingge Scientific Instruments Co., Ltd., Hangzhou, China). The fused fragment was transformed into fungal protoplasts using polyethylene glycol (PEG)-mediated protoplast transformation. Putative *FgSRE1* deletion mutants (Δ*FgSRE1*) were screened on regeneration medium supplemented with hygromycin B (100 μg/mL), and positive mutants were verified by PCR genotyping (Figure S1) [26]. A total of twelve independent deletion transformants were obtained and screened by PCR. Three independent mutants (Δ*FgSRE1*-1, Δ*FgSRE1*-2, and Δ*FgSRE1*-3) were selected for phenotypic assays, all of which displayed consistent phenotypic defects. This confirmed that the observed phenotypes were not due to ectopic integration events.

**Figure 1:**
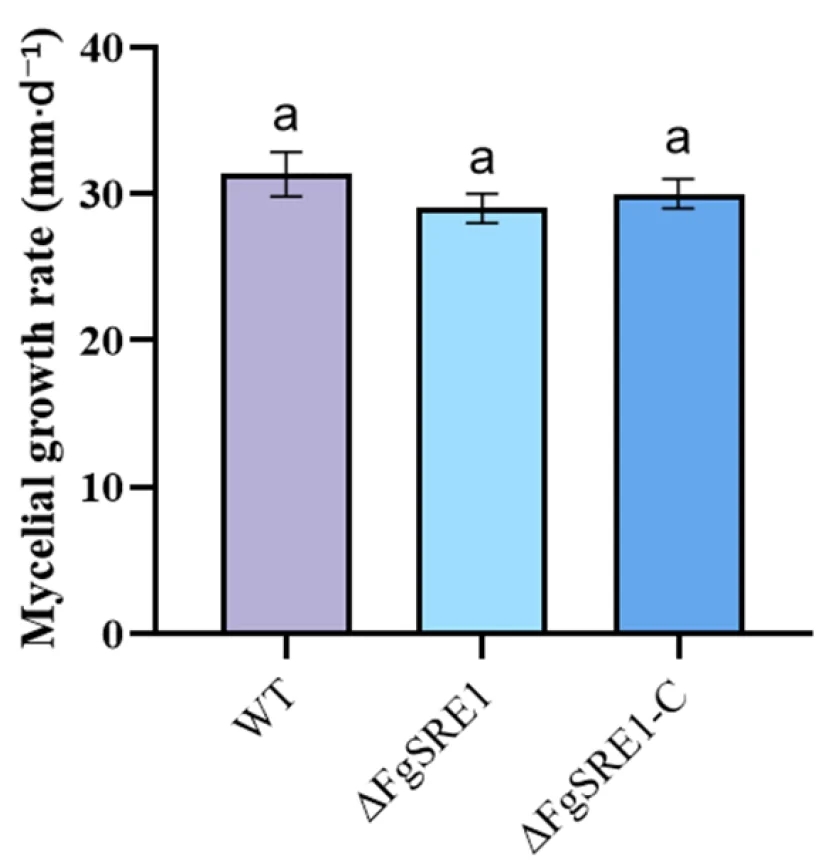
Radial growth rates of WT, Δ*FgSRE1*, and Δ*FgSRE1*-C strains. The radial growth rates were measured after 3 days of incubation on PDA at 25 °C. Data are presented as mean ± SD (*n* = 3 biological replicates). Data were analyzed using one-way ANOVA. The same letter above the bars indicates no significant difference (*p* > 0.05).

**Figure 2:**
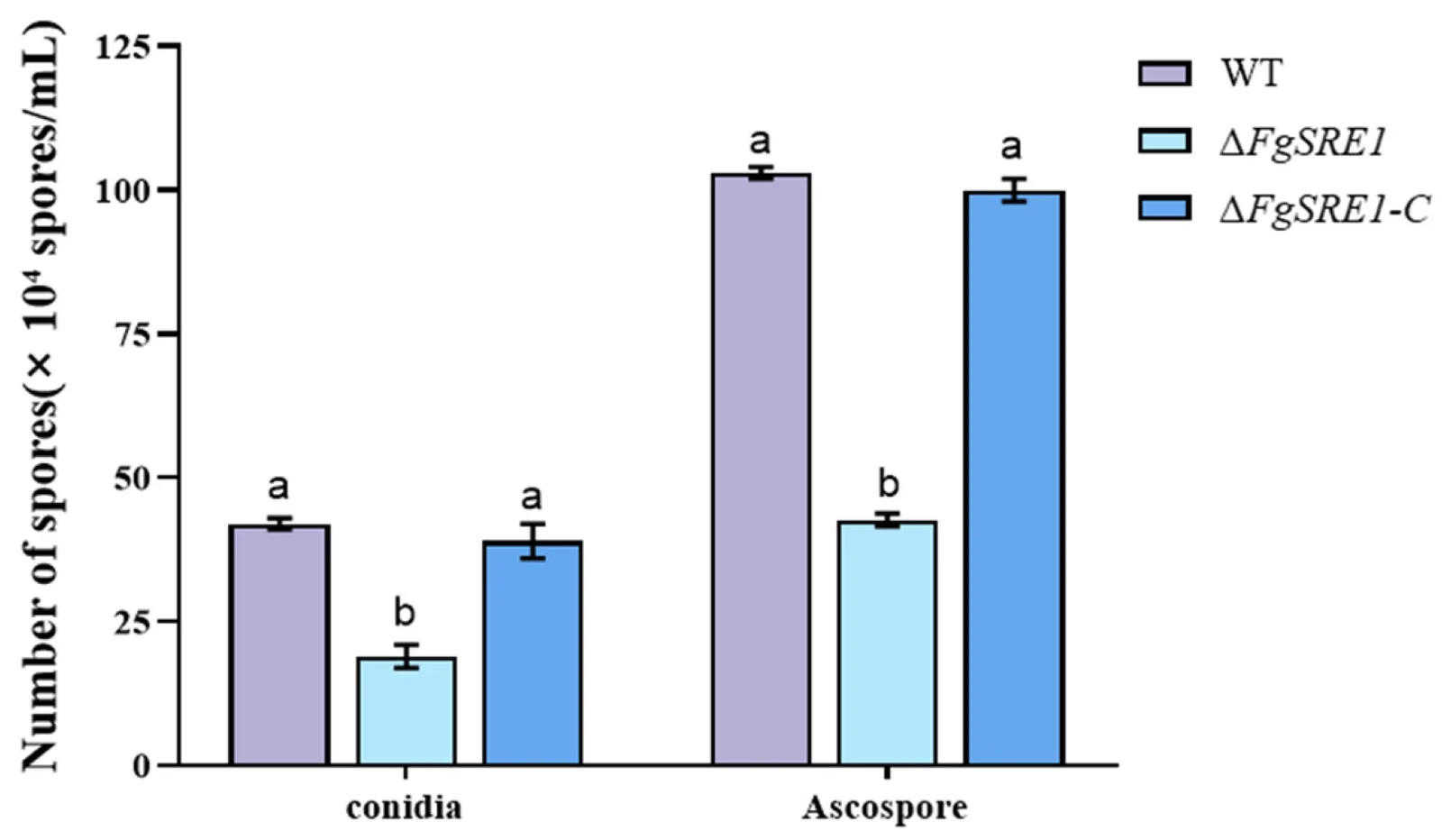
Quantitative analysis of asexual and sexual spore production. The bar chart compares conidia and ascospore yields of *WT*, Δ*FgSRE1*, and Δ*FgSRE1*-C strains. Data were analyzed using one-way ANOVA followed by Tukey’s HSD post-hoc test for multiple comparisons. The vertical axis indicates spore abundance with the unit of ×10^4^ spores/mL. WT, wild-type strain; Δ*FgSRE1*, *FgSRE1* knockout mutant; Δ*FgSRE1*-C, complemented strain. All data are presented as the mean ± standard deviation (SD) (*n* = 3 biological replicates per strain). Different letters above the bars indicate statistically significant differences (*p* < 0.05); The same letter above the bars indicates no significant difference (*p* > 0.05).

**Figure 3:**
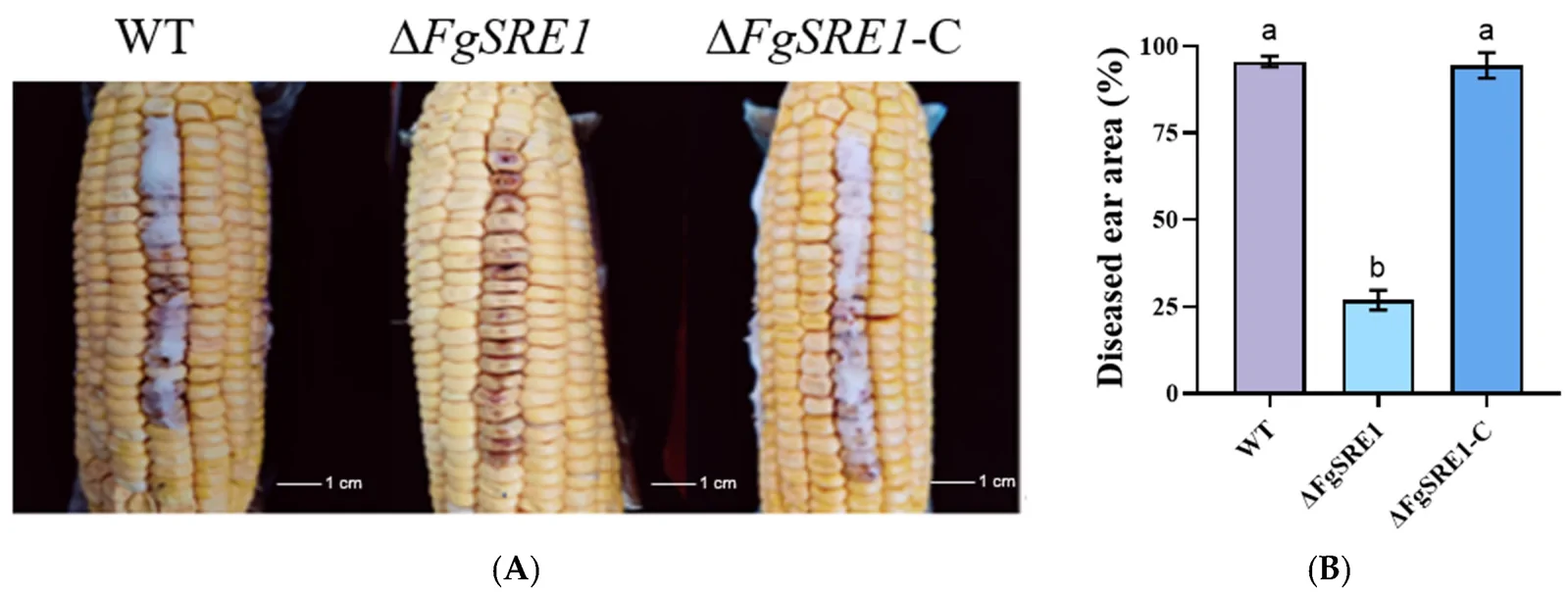
Pathogenicity of Δ*FgSRE1* on maize ears. Maize ears were divided into three groups and inoculated with spore suspensions of the wild-type (WT), Δ*FgSRE1* mutant, and Δ*FgSRE1-C* strain. All ears were sealed and incubated at 25 °C for 2 days, after which lesion areas within the inoculated region (two rows of kernels per ear) were measured using ImageJ (version 1.54s; NIH, Bethesda, MD, USA). (**A**) Representative images of diseased maize ears inoculated with the WT, Δ*FgSRE1* mutant, and Δ*FgSRE1-C* strain. (**B**) Quantification of diseased within the inoculated region (%). Data are presented as mean ± SD (*n* = 3 ears per group, representing three biological replicates). Data were analyzed using one-way ANOVA followed by Tukey’s HSD post-hoc test for multiple comparisons. Different letters above the bars indicate statistically significant differences (*p* < 0.05).

**Figure 4:**
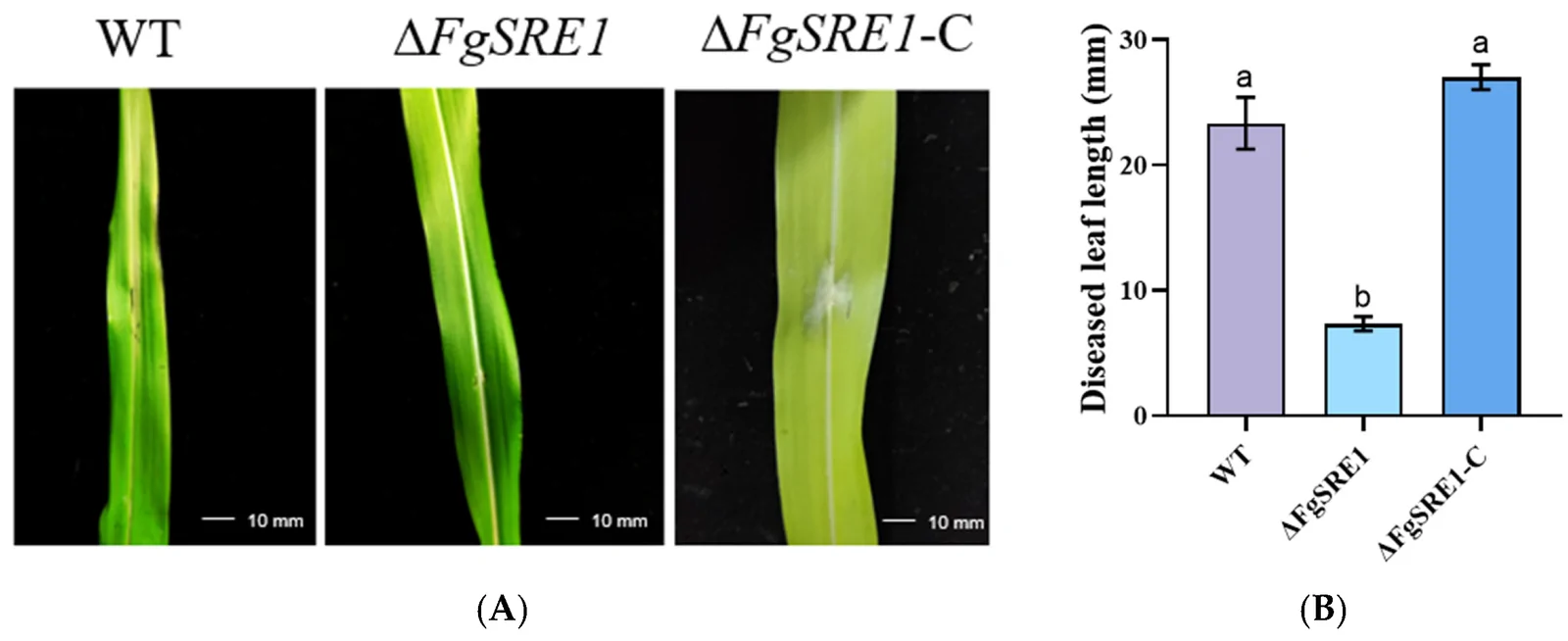
Pathogenicity of Δ*FgSRE1* on maize leaves. Maize leaves were inoculated with spore suspensions of WT, Δ*FgSRE1* and Δ*FgSRE1*-C. Following incubation, diseased leaf lengths were measured. (**A**) Representative infection phenotypes on maize leaves for WT, Δ*FgSRE1* and Δ*FgSRE1*-C. (**B**) Quantification of diseased leaf length (mm). Data are presented as mean ± SD (*n* = 5 leaves per biological replicate; three independent biological replicates were performed on three different dates, totaling 15 leaves per treatment). Data were analyzed using one-way ANOVA followed by Tukey’s HSD post hoc test for multiple comparisons. Different letters above the bars indicate statistically significant differences (*p* < 0.05).

To construct the complemented strain, the open reading frame of *FgSRE1* together with its endogenous promoter region was amplified by PCR and seamlessly cloned into the pBLUE-GFP vector carrying G418 resistance (200 μg/mL). The recombinant plasmid was transformed into *Escherichia coli*, and positive clones were confirmed by Sanger sequencing to obtain the complementation vector. The complementation construct was designed to integrate at the native *FgSRE1* locus through homologous recombination, replacing the deleted gene with the full-length *FgSRE1* coding sequence under the control of its native promoter. The sequence-verified plasmid was introduced into the Δ*FgSRE1* mutant via PEG-mediated protoplast transformation [27]. Transformants were selected on G418-containing medium, and the resulting complemented strain Δ*FgSRE1*-C was further authenticated by GFP fluorescence observation (Figure S2) and, more importantly, by complete phenotypic restoration to wild-type levels in all assays (Figure 1, Figure 2, Figure 3 and Figure 4). The GFP signal confirms expression of the fusion construct in the complemented fungal strain but does not demonstrate secretion into plant tissue.

### 2.3. Phenotypic Characterization of the ΔFgSRE1 Mutant

Mycelial plugs of WT, Δ*FgSRE1* mutant, and Δ*FgSRE1*-C were inoculated onto PDA plates and incubated at 25 °C for 3 days. Colony morphology and radial growth were recorded daily; radial mycelial diameters were measured via the cross method using two perpendicular lines through the colony center for average calculation. For conidiation assays, three equal-sized mycelial plugs (5 mm diameter) were cultured in conical flasks containing 50 mL of CMC liquid medium on a rotary shaker (SKY-200B; SUKUN, Shanghai, China) at 175 rpm at 25 °C for 3 days. The cultures were filtered through three layers of Miracloth (Shanghai Jinpan Biotechnology Co., Ltd., Shanghai, China), and the filtrate was collected. The filtrate was centrifuged at 5000 rpm for 10 min using a high-speed centrifuge (HC-3518; Anhui Zhongke Zhongjia Scientific Instruments Co., Ltd., Hefei, China), and the conidial pellets were resuspended in 3 mL of sterile distilled water. Conidia were counted with a hemocytometer (Shanghai Qiujing Biochemical Reagent & Instrument Co., Ltd., Shanghai, China) under an optical microscope (Ningbo Yongxin Optics Co., Ltd., Ningbo, China). For each sample, conidia in five hemocytometer squares (1 × 1 × 0.1 mm) were counted, and the average was calculated. Conidial production was expressed as the number of conidia per mL of suspension. Three technical replicates were performed for each biological replicate. To induce sexual development, mycelial plugs were placed on CA medium and incubated statically at 25 °C for 5 days until full colony coverage. Subsequently, 1 mL of 0.1% Tween-20 solution was added, and the mycelia were pressed flat using a sterile spatula. The cultures were incubated for another 7 days at 25 °C with a photoperiod of 12 h darkness followed by 12 h black light prior to ascospore enumeration using a hemocytometer under microscopy [28]. Ascospore production was expressed as ascospores per mL. All experiments were performed with three independent biological replicates for each sample to guarantee accuracy and repeatability.

### 2.4. Regulatory Roles of FgSRE1 in Pathogenicity of Fusarium graminearum

Maize ears of the susceptible cultivar “Xianyu 335” (*Zea mays* L.) at the milk stage were collected from plants grown in the experimental field of Yan’an University. The ears were surface-sterilized with 75% ethanol for 3 min, rinsed three times with sterile distilled water, and air-dried in a laminar flow hood before inoculation. The ears were detached from the plants and placed on sterile moist filter paper in trays to maintain humidity. Nine sterilized maize ears were equally divided into three groups. Each kernel in two rows on every ear was injected with 5 μL 1 × 10^5^ CFU/mL spore suspensions of WT, Δ*FgSRE1* mutant, and Δ*FgSRE1*-C complemented strain using a sterile syringe (Shandong Weigao Group Medical Polymer Co., Ltd., Weihai, China). All ears were sealed in plastic bags and incubated at 25 °C in a controlled-environment incubator for 2 days for lesion area measurement. The inoculated area of each ear (defined as the region containing the two injected rows of kernels) was photographed, and the diseased area within this region was quantified using ImageJ software (version 1.54s; NIH, Bethesda, MD, USA). The percentage of diseased area was calculated as (diseased area/total inoculated area) × 100%. The biological experimental unit was defined as an individual ear.

One-month-old maize seedlings were wounded at the leaf midpoint with sterile needles. Sterile filter strips soaked in corresponding spore suspensions were wrapped around the wounds and covered with plastic wrap for moisture retention. Seedlings were cultured in a light incubator (LRH-500F; Shanghai Qixin Scientific Instruments Co., Ltd., Shanghai, China) at 25 °C light / 22 °C dark for 2 days to quantify lesion lengths. The biological experimental unit was defined as an individual leaf. Three independent biological replicates (each comprising five leaves) were performed for each treatment. All experiments were repeated independently on three different dates. Lesion measurements were performed blinded to treatment by coding the samples prior to analysis.

### 2.5. Preparation of Samples for Transcriptome Sequencing

Mycelial plugs of WT and Δ*FgSRE1* mutants were cultured on PDA medium at 25 °C in darkness for 3 days. Mycelia of the purely cultured WT (Fg) and Δ*FgSRE1* (TBRFg) without host treatment were collected as control groups. For infection groups, sterile maize ears were placed onto fungal colonies and incubated under identical conditions for another 2 days. Subsequently, wild-type mycelia (FgHZ) were harvested from maize ears colonized by WT, and Δ*FgSRE1* mycelia (TBRFgHZ) were harvested from maize ears colonized by the Δ*FgSRE1* mutant. All samples were stored at −80 °C. Each treatment was performed in three biological replicates, and the samples were submitted to Jizhijiyin Gene Biotechnology Co., Ltd. (Qingdao, China) for transcriptome sequencing.

### 2.6. RNA Extraction, Library Construction, and Sequencing

Total RNA was extracted from all prepared samples using TRIzol reagent (Invitrogen, Carlsbad, CA, USA) according to the manufacturer’s protocol. RNA integrity and concentration were evaluated using an Agilent 2100 Bioanalyzer (Agilent Technologies, Santa Clara, CA, USA), and only samples with RNA integrity number (RIN) ≥ 8.0 were used for subsequent library construction. mRNA was enriched with oligo (dT) magnetic beads, fragmented, and used for stranded mRNA library construction following the manufacturer’s instructions of the NEBNext^®^ Ultra™ II RNA Library Prep Kit for Illumina^®^ (NEB, Ipswich, MA, USA). The libraries were sequenced on the Illumina NovaSeq 6000 platform (Illumina, San Diego, CA, USA), generating 150 bp paired-end reads.

### 2.7. RNA-Seq Data Processing and Analysis

Raw reads were quality-filtered with fastp (v0.23.2) with default parameters to obtain clean reads, which were mapped to the *F. graminearum* reference genome (GCF_000240135.3_ASM24013v3_genomic.fna) using HISAT2 (version 2.2.1) with default parameters [29]. Transcripts were assembled via StringTie (version 3.0.3), and gene expression levels were quantified using featureCounts (version 2.0.1) with the annotation file (GCF_000240135.3_ASM24013v3_genomic.gtf). Only uniquely mapped reads were retained for quantification; multimapping reads were excluded. Raw integer read counts generated by featureCounts were used as input for DESeq2 (version 1.42.0) [30]; FPKM values were calculated separately for visualization purposes only. Pearson correlation analysis was performed to assess the reproducibility of biological replicates. Differentially expressed genes (DEGs) were identified by DESeq2 [30] with the threshold of padj < 0.05 and |log_2_(fold change)| ≥ 1. GO enrichment and KEGG pathway enrichment analyses were subsequently carried out.

Two pairwise transcriptome comparisons were set up: Fg vs. TBRFg to explore *FgSRE1*-dependent gene regulation during host-free vegetative growth, and FgHZ vs. TBRFgHZ to investigate the regulatory function of *FgSRE1* during host infection.

### 2.8. Validation via Quantitative Real-Time PCR

Nine core DEGs linked to transmembrane transport, membrane homeostasis, and oxidative stress pathways, including four upregulated genes encoding transferases and five downregulated transporter genes, were selected for qRT-PCR to validate transcriptome reliability. For qRT-PCR validation, mycelia were collected at six time points (0, 12, 24, 36, 48, and 72 h) post-inoculation of maize ears with *F. graminearum*. Total RNA was extracted using TRIzol reagent (Invitrogen, Carlsbad, CA, USA), and cDNA was synthesized using the PrimeScript™ RT Reagent Kit with gDNA Eraser (Takara, Kyoto, Japan). qRT-PCR was performed using TB Green^®^ Premix Ex Taq™ II (Tli RnaseH Plus) (Takara, Kyoto, Japan) on a LightCycler^®^ 480 Real-Time PCR system (Roche, Basel, Switzerland). Specific primers were designed with Primer Premier (version 5.0; Premier Biosoft, Palo Alto, CA, USA), and all sequences are provided in Table S2. The endogenous Tubulin gene was used as the internal reference for normalization. Relative expression was calculated by the 2^−ΔΔCT^ method. Three technical replicates were performed for each biological replicate. Primer efficiencies were confirmed by standard curve analysis, and melt-curve analysis confirmed the specificity of each primer pair. Statistical significance was determined using one-way ANOVA followed by Dunnett’s post hoc test, with the 0 h time point as the control. All experiments were performed with three independent biological replicates for each sample to guarantee accuracy and repeatability.

## 3. Results

### 3.1. FgSRE1 Is Dispensable for Vegetative Growth of Fusarium graminearum

We incubated WT, Δ*FgSRE1*, and complemented Δ*FgSRE1*-C strains on solid medium for 3 days and quantified colony diameters. The radial growth rates of WT, Δ*FgSRE1*, and Δ*FgSRE1*-C were 31.3 ± 0.3 mm·d^−1^, 29.0 ± 0.2 mm·d^−1^, and 30.0 ± 0.3 mm·d^−1^, respectively, without any statistically significant difference. Collectively, deletion of *FgSRE1* does not affect vegetative growth of *F. graminearum*.

### 3.2. FgSRE1 Contributes to Reproductive Development in Fusarium graminearum

We quantified conidial and ascospore yields of the wild-type (WT), Δ*FgSRE1* mutant, and Δ*FgSRE1*-C complemented strain. In asexual sporulation assays, conidial production of Δ*FgSRE1* was markedly reduced. Compared with WT and Δ*FgSRE1*-C, the mutant displayed 54.4% and 50.9% lower conidiation, respectively (*p* < 0.0001; Figure 2). For sexual reproduction, ascospore production of Δ*FgSRE1* decreased by 59.7% and 58% relative to WT and Δ*FgSRE1*-C, respectively (*p* < 0.0001; Figure 2). The Δ*FgSRE1* mutant showed a 54.4% reduction in conidial yield and a 59.7% reduction in ascospore yield compared with WT. At the same time, the sporulation ability of Δ*FgSRE1*-C was fully restored to the wild-type level with no significant difference (ns).

Collectively, targeted deletion of *FgSRE1* severely impairs both asexual and sexual sporulation in *F*. *graminearum*. These results reveal that *FgSRE1* acts as a vital regulator of asexual reproduction and sexual development.

### 3.3. The Effect of FgSRE1 on the Pathogenicity of Fusarium graminearum

Nine maize ears were divided into three groups and inoculated with spore suspensions (5 μL, 1 × 10^5^ CFU/mL) of the WT, Δ*FgSRE1* mutant, and Δ*FgSRE1*-C strain. All ears were sealed and incubated at 25 °C for 2 days, after which lesion areas were measured. As shown in Figure 3, the diseased area proportions of WT and Δ*FgSRE1*-C reached 95.56% and 94.44%, respectively, showing no obvious difference between the two strains. In contrast, the Δ*FgSRE1* mutant only exhibited a diseased area of 26.86%, indicating markedly attenuated virulence compared with the wild-type and complemented strains. These results demonstrate that *FgSRE1* is critical for full virulence of *F. graminearum* during infection of maize ears.

Fifteen maize leaves were inoculated with spore suspensions (5 μL, 1 × 10^5^ CFU/mL) of the WT, Δ*FgSRE1* mutant, and Δ*FgSRE1*-C strain. All leaves were cultured in a light incubator (25 °C light/22 °C dark) for 2 days to measure lesion lengths. As shown in Figure 4, Δ*FgSRE1* induced markedly shorter lesions than WT and Δ*FgSRE1*-C, and a significant difference was also observed between WT and Δ*FgSRE1*-C, revealing severely weakened virulence of the mutant. These results demonstrate that *FgSRE1* is critical for full virulence of *F. graminearum* during infection of maize leaves.

### 3.4. RNA Quality Control Analysis

Twelve RNA samples, including Fg_1/2/3, FgHZ_1/2/3, TBRFg_1/2/3, and TBRFgHZ_1/2/3, were examined using an Agilent 2100 Bioanalyzer. All samples were eligible for library preparation and sequencing.

### 3.5. Quality Assessment of Sequencing Data

Raw sequencing reads were filtered to obtain clean reads. Detailed quality parameters of clean reads are listed in Table 1.

Sequencing data showed high consistency across four sample groups. The valid data size was 5.83 G (wild-type pure culture, Fg), 6.03 G (wild-type infection, FgHZ), 5.93 G (mutant pure culture, TBRFg), and 6.08 G (mutant infection, TBRFgHZ). The effective data rates (clean data/raw data) were over 93% for most libraries, except for TBRFgHZ_1 (74%). The lower effective rate of TBRFgHZ_1 was likely due to the presence of host plant tissue debris during mycelium harvesting from infected maize ears, which introduced additional plant-derived sequences that were removed during quality filtering. Importantly, the clean reads of this sample (5.32 G) were sufficient for downstream analyses, and the uniquely mapped reads to the fungal reference genome exceeded 85% (Table 2), confirming that the majority of reads were of fungal origin. The global error rate remained at 0.02%. The Q20 and Q30 scores exceeded 98.00% and 94.00%, respectively, and rRNA contamination was at an extremely low level (rRNA ratios ranging from 0.10% to 0.90%; Table 1).

**Figure 5:**
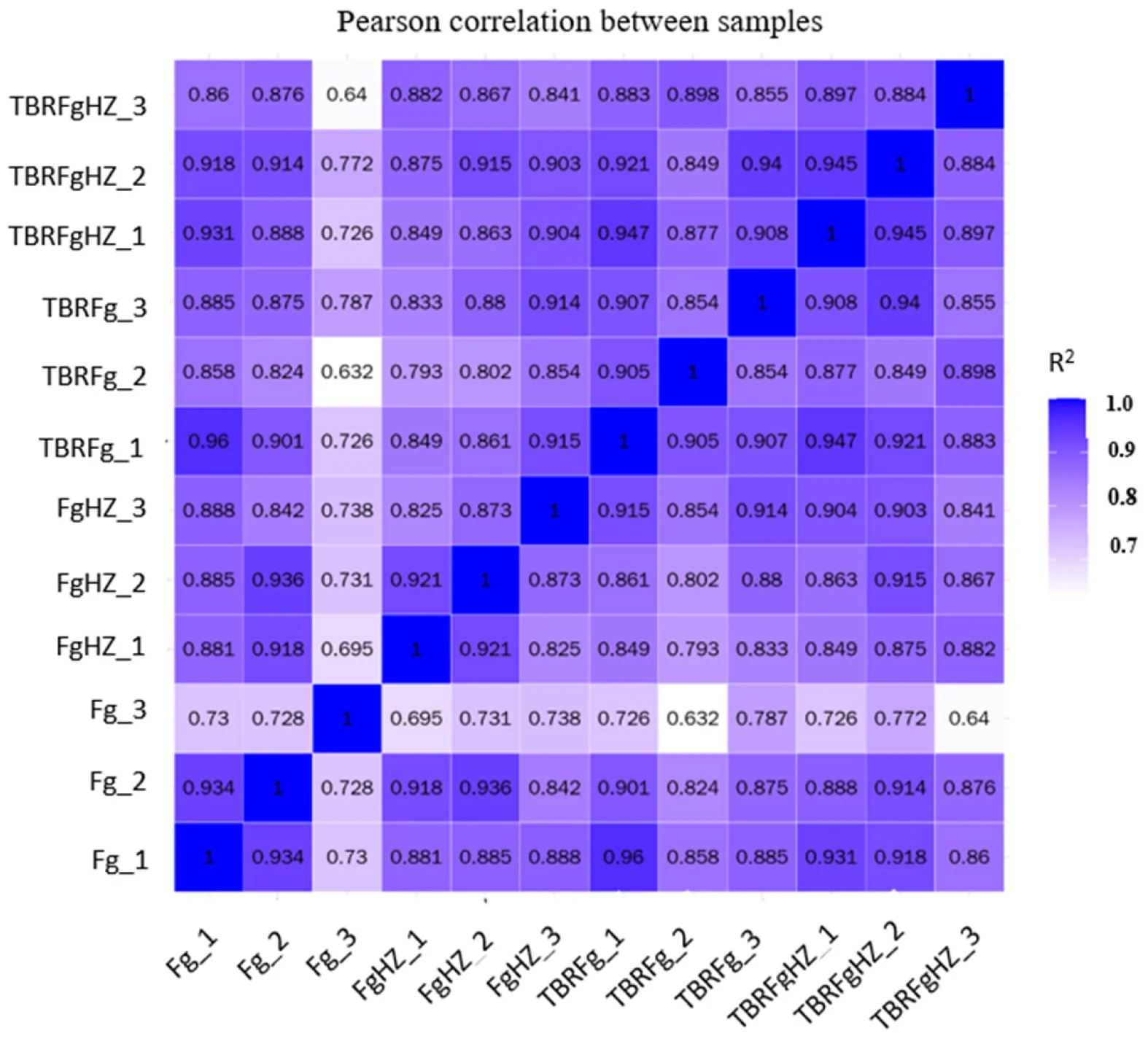
Pearson correlation analysis of transcriptome samples. The heatmap displays the squared Pearson correlation coefficient (R2) between every pair of samples. Color gradient: light purple (R^2^ = 0.7) to dark blue (R^2^ = 1.0) indicates increasing correlation strength. *n* = 3 biological replicates per group. Fg_1/2/3: three biological replicates of wild-type *F. graminearum* pure culture; FgHZ_1/2/3: three biological replicates of wild-type hyphae recovered from infected maize ears; TBRFg_1/2/3: three biological replicates of Δ*FgSRE1* pure culture; TBRFgHZ_1/2/3: three biological replicates of Δ*FgSRE1* hyphae recovered from infected maize ears.

Pearson correlation analysis was performed to evaluate the reproducibility of biological replicates. A coefficient (R^2^) approaching 1 represents a higher correlation, and values greater than 0.8 indicate a strong correlation. To assess reproducibility, we focused on the correlations among biological replicates within each treatment group. Within-group R^2^ values were as follows: Fg group (0.89–0.94), FgHZ group (0.85–0.92), TBRFg group (0.82–0.91), and TBRFgHZ group (0.81–0.90). Figure 5 shows that all pairwise R^2^ values for gene expression were over 0.64, with the majority above 0.8. Collectively, the samples were reliable, and the sequencing datasets exhibited good reproducibility.

Clean reads from twelve samples were aligned against the *F. graminearum* reference genome to assess data reliability (Table 2). Mapping efficiency was consistent across all experimental groups. The average mapping rates were 88.65% (Fg_1/2/3, wild-type pure culture), 87.94% (FgHZ_1/2/3, wild-type infection), 86.30% (TBRFg_1/2/3, mutant pure culture), and 86.63% (TBRFgHZ_1/2/3, mutant infection). The mapping rate of each sample varied from 85.14% to 93.12%, and uniquely mapped reads predominated over multiply mapped reads. These results demonstrate that the obtained sequencing data were of high quality and could be accurately mapped to the reference genome. The reference genome is therefore appropriate for downstream gene expression quantification and differential gene analysis.

### 3.6. Analysis of Differentially Expressed Genes

**Figure 6:**
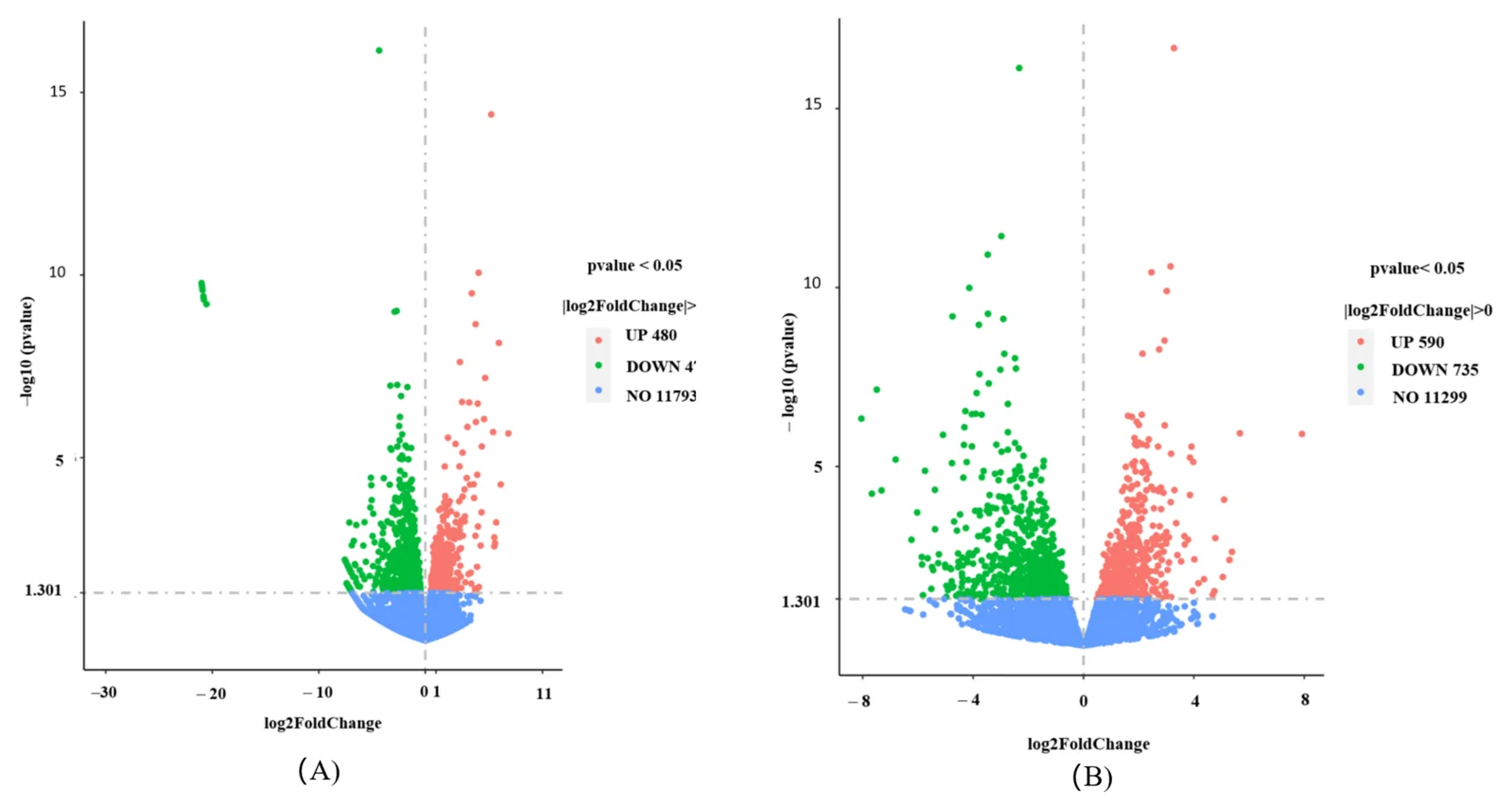
Volcano plots of differentially expressed genes. Panels (**A**,**B**) show volcano plots for DEGs from the TBRFg vs. Fg and TBRFgHZ vs. FgHZ comparisons, respectively. DEGs were screened under thresholds |log_2_ (fold change)| ≥ 1 and padj < 0.05. *n* = 3 biological replicates per group. In panel (**A**), red dots represent 480 significantly upregulated genes, green dots indicate 749 significantly downregulated genes, and blue dots correspond to 11,793 genes with non-significant expression changes. In panel (**B**), red dots represent 590 significantly upregulated genes, green dots indicate 735 significantly downregulated genes, and blue dots correspond to 11,299 genes with non-significant expression changes.

Differentially expressed genes (DEGs) were filtered using the thresholds |log_2_ (fold change)| ≥ 1 and padj < 0.05. The distribution profiles of DEGs from the two pairwise comparisons are presented as volcano plots in Figure 6. As summarized in Table 3, in the TBRFg vs. Fg comparison, 1229 DEGs were identified, including 480 significantly upregulated and 749 downregulated genes. For the TBRFgHZ vs. FgHZ comparison, 1325 DEGs were detected, with 590 upregulated and 735 downregulated transcripts.

**Table 3:** Statistical Table on the number of differential genes.

Pairs	Up	Down	Total
TBRFg vs. Fg	480	749	1229
TBRFgHZ vs. FgHZ	590	735	1325

Note: Pairs: A_VS_B represents a comparison between A (experimental group) and B (control group); Up: Number of upregulated genes, i.e., genes that are upregulated in A relative to B; Down: Number of downregulated genes, i.e., genes that are downregulated in A relative to B; Total: Total number of differentially expressed genes.

**Figure 7:**
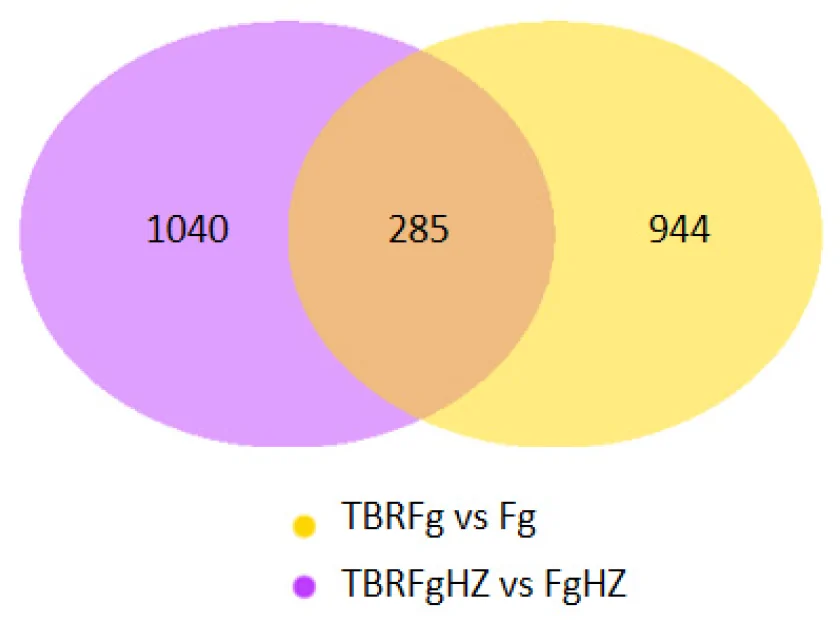
Venn diagram of DEGs shared between the two comparison groups. The yellow circle stands for DEGs unique to TBRFg vs. Fg (944 genes), the purple circle represents DEGs detected only in the TBRFgHZ vs. FgHZ comparison (1040 genes), and their overlapping region contains 285 common DEGs.

To identify DEGs potentially associated with *FgSRE1* function during maize ear rot infection and to exclude genes that are differentially expressed under axenic conditions, Venn diagram analysis was performed on the two DEG datasets (Figure 7). A total of 944 DEGs were unique to the TBRFg vs. Fg group, 1040 DEGs were detected only in the TBRFgHZ vs. FgHZ comparison, and 285 DEGs were shared between both groups. The 1040 DEGs uniquely detected in the infection comparison suggest that *FgSRE1* deletion may trigger extensive transcriptional reprogramming in *F. graminearum* upon host colonization. The 285 overlapping DEGs were only counted for Venn statistics, rather than selected for separate functional enrichment analysis to further dissect the downstream regulatory networks of *FgSRE1*.

### 3.7. Gene Ontology Enrichment Analysis

**Figure 8:**
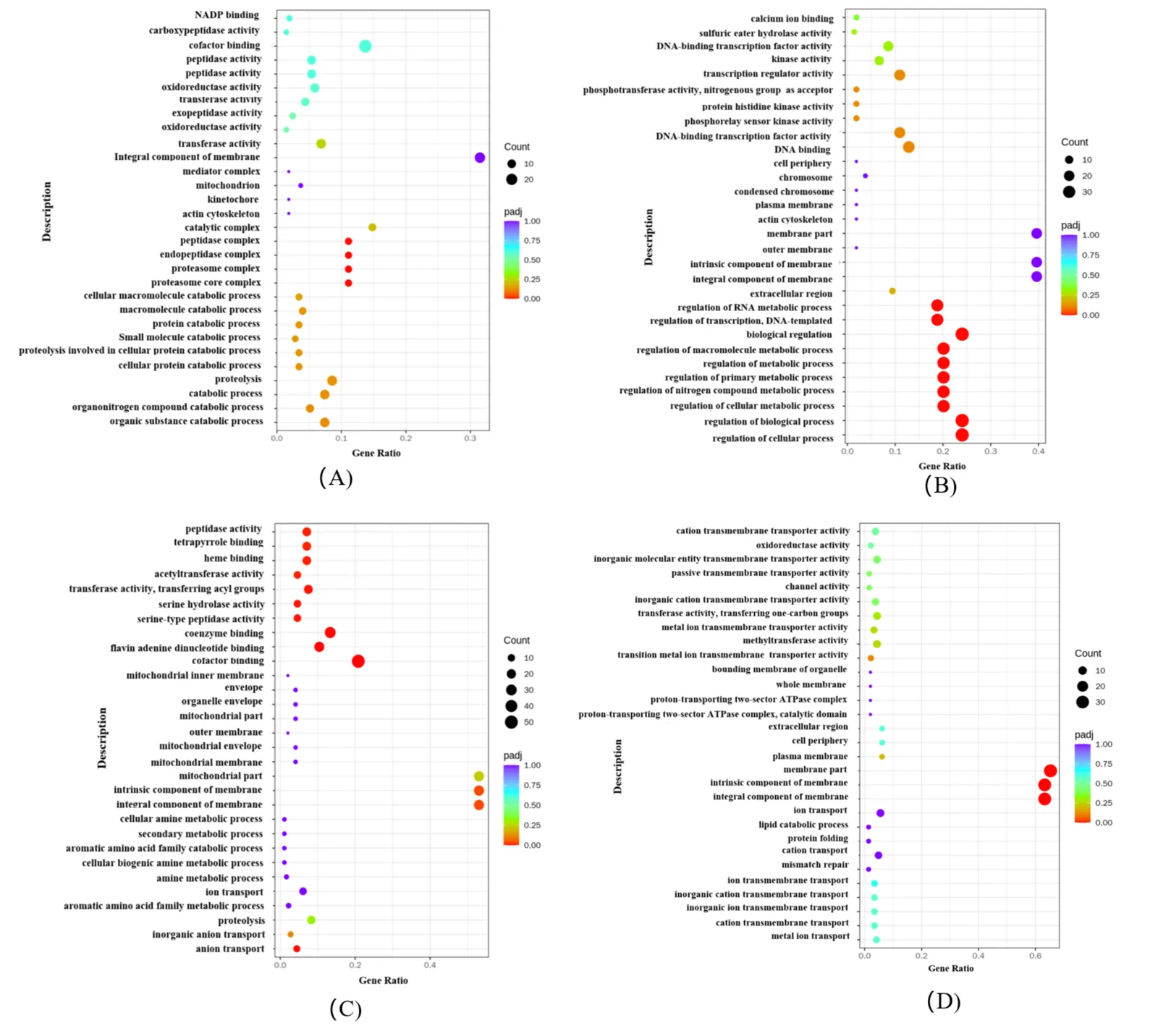
GO functional enrichment analysis of differentially expressed genes. Scatter plots showing the top enriched Gene Ontology (GO) terms among upregulated and downregulated DEGs from each pairwise comparison. (**A**) Upregulated DEGs in the TBRFg vs. Fg comparison (axenic culture; 480 genes; *n * = 3 biological replicates per group); (**B**) downregulated DEGs in the TBRFg vs. Fg comparison (axenic culture; 749 genes); (**C**) upregulated DEGs in the TBRFgHZ vs. FgHZ comparison (maize ear infection; 590 genes); (**D**) downregulated DEGs in the TBRFgHZ vs. FgHZ comparison (maize ear infection; 735 genes). The *x*-axis represents the Gene Ratio (the proportion of DEGs annotated to a given GO term relative to the total number of DEGs in the analyzed gene set), the *y*-axis indicates the GO terms, and the color scale represents the adjusted *p*-value (padj). The size of each dot reflects the number of DEGs (Count) annotated to the corresponding GO term. GO terms are categorized into biological process (BP), cellular component (CC), and molecular function (MF). Separate enrichment of up/down DEGs; background: all annotated PH-1 protein-coding genes.

To preserve the directionality of transcriptional responses, GO enrichment analysis was performed separately for upregulated and downregulated DEGs from each pairwise comparison (Figure 8). For TBRFg vs. Fg (480 up, 749 down) and TBRFgHZ vs. FgHZ (590 up, 735 down), all enrichment adopted all annotated protein-coding genes of the *F. graminearum* PH-1 reference genome as background.

Under axenic conditions (TBRFg vs. Fg), upregulated DEGs in the Δ*FgSRE1* mutant were significantly enriched in proteolysis and protein catabolic processes, with associated molecular functions including peptidase activity, carboxypeptidase activity, and oxidoreductase activity (Figure 8A). Cellular component terms were predominantly assigned to the proteasome complex and catalytic complex, indicating enhanced protein degradation in the mutant. In contrast, downregulated DEGs were enriched in regulation of transcription and RNA metabolic processes, with molecular functions dominated by calcium ion binding, DNA-binding transcription factor activity, and kinase activity (Figure 8B). Cellular component analysis revealed enrichment in membrane components and the plasma membrane, suggesting reduced transcriptional regulatory capacity and potential membrane-related defects.

Under infection conditions (TBRFgHZ vs. FgHZ), upregulated DEGs were significantly enriched in ion transport, amine metabolic process, and secondary metabolism (Figure 8C). Molecular functions were dominated by heme binding, tetrapyrrole binding, and cofactor binding, while cellular components were mainly assigned to mitochondrial membrane and membrane components. This suggests that the mutant may activate mitochondrial functions and secondary metabolism during host infection. Downregulated DEGs under infection conditions were enriched in ion transport and cation transmembrane transport, with molecular functions including cation transmembrane transporter activity, channel activity, and methyltransferase activity (Figure 8D). Cellular components were predominantly assigned to plasma membrane and membrane parts, indicating that membrane-associated transport functions are compromised in the mutant during infection.

Collectively, the GO enrichment analysis suggests that *FgSRE1* deletion may lead to compensatory activation of proteolysis under axenic conditions, while transcriptional changes in membrane-associated transport and transcriptional regulation are observed. These transcriptomic alterations are further pronounced during host infection, particularly affecting genes involved in mitochondrial function and ion transport pathways. The possible effects on membrane integrity, oxidative-stress tolerance, and mycotoxin production require direct experimental validation.

### 3.8. KEGG Pathway Enrichment Analysis

**Figure 9:**
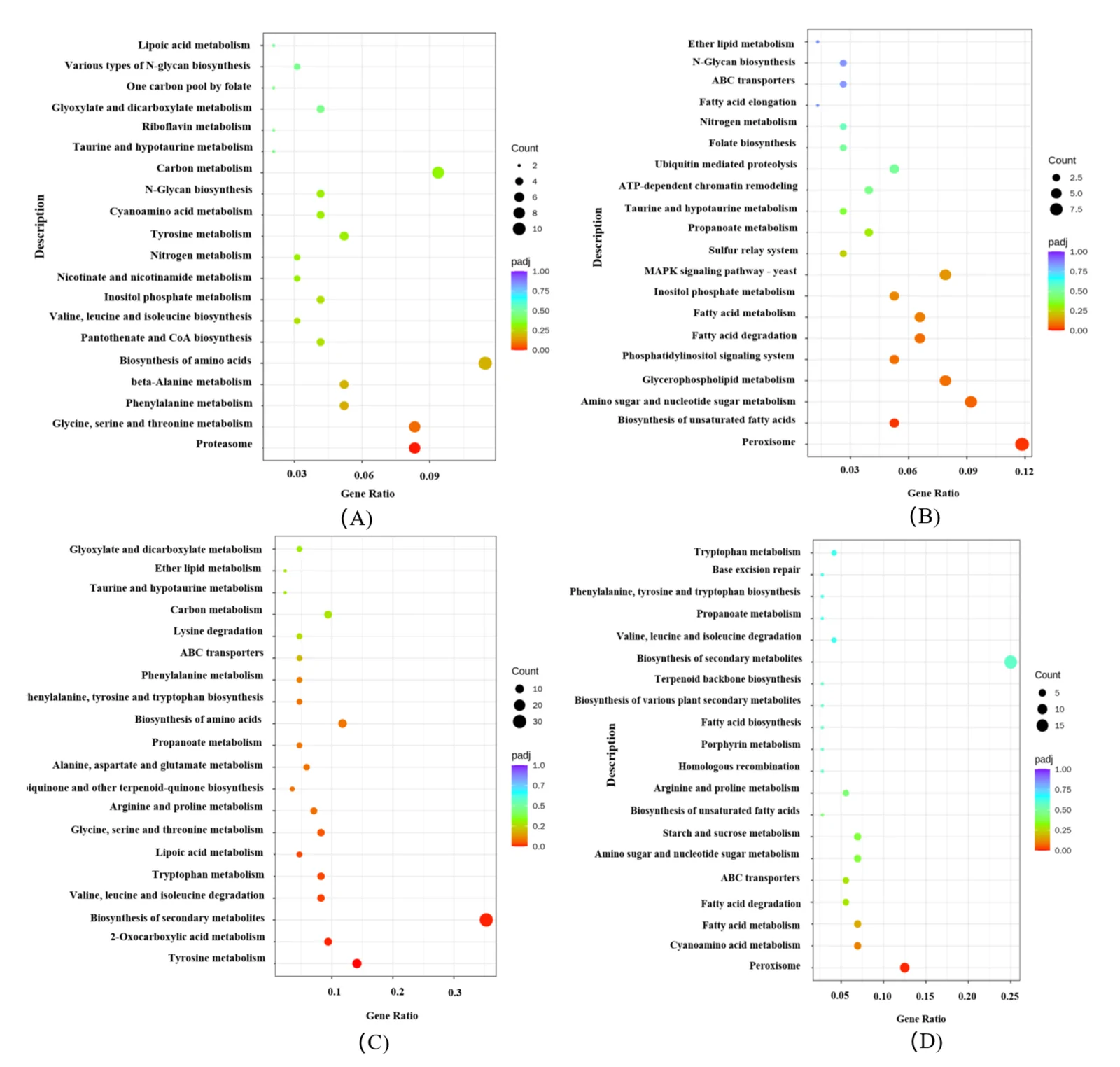
KEGG pathway enrichment analysis of differentially expressed genes. Scatter plots showing the top enriched KEGG pathways among upregulated and downregulated DEGs from each pairwise comparison. (**A**) Upregulated DEGs in the TBRFg vs. Fg comparison (axenic culture; 480 genes; *n* = 3 biological replicates per group); (**B**) downregulated DEGs in the TBRFg vs. Fg comparison (axenic culture; 749 genes); (**C**) upregulated DEGs in the TBRFgHZ vs. FgHZ comparison (maize ear infection; 590 genes); (**D**) downregulated DEGs in the TBRFgHZ vs. FgHZ comparison (maize ear infection; 735 genes). The *x*-axis represents the Gene Ratio (the proportion of DEGs annotated to a given pathway relative to the total number of DEGs in the analyzed gene set), the *y*-axis indicates the KEGG pathway terms, and the color scale represents the adjusted *p*-value (padj). The size of each dot reflects the number of DEGs (Count) annotated to the corresponding pathway. Separate enrichment of up/down DEGs; background: all annotated PH-1 protein-coding genes.

KEGG pathway enrichment analysis was performed separately for upregulated and downregulated DEGs from each pairwise comparison to preserve the directionality of pathway-level responses (Figure 9). The same gene set and background criteria described above were used for all KEGG enrichment.

Under axenic conditions (TBRFg vs. Fg), upregulated DEGs in the Δ*FgSRE1* mutant were significantly enriched in one-carbon pool by folate, glyoxylate and dicarboxylate metabolism, N-glycan biosynthesis, and proteasome pathways (Figure 9A). In contrast, downregulated DEGs were enriched in peroxisome, glycerophospholipid metabolism, fatty acid metabolism, fatty acid degradation, and ABC transporters (Figure 9B). These results suggest that FgSRE1 deletion may trigger compensatory activation of metabolic pathways related to amino acid metabolism and protein degradation, while transcriptional suppression of lipid metabolism and peroxisomal pathway-associated genes is observed in the mutant under axenic conditions.

Under infection conditions (TBRFgHZ vs. FgHZ), upregulated DEGs were significantly enriched in biosynthesis of secondary metabolites, amino acid biosynthesis, glyoxylate and dicarboxylate metabolism, and tyrosine metabolism (Figure 9C). Notably, ABC transporters also appeared in the upregulated gene set, suggesting that certain transport-related genes may be activated during host infection. Downregulated DEGs under infection conditions were enriched in peroxisome, fatty acid degradation, ABC transporters, and tryptophan metabolism (Figure 9D). The consistent enrichment of peroxisome and fatty acid degradation among downregulated genes under both conditions suggests that *FgSRE1* deletion may be associated with persistent transcriptional changes in peroxisomal and lipid metabolic pathways, which appear more pronounced during host infection. The possible effects on membrane integrity, oxidative-stress tolerance, and mycotoxin production require direct experimental validation.

### 3.9. A Proposed Model for FgSRE1-Mediated Positive Regulation of Virulence

**Figure 10:**
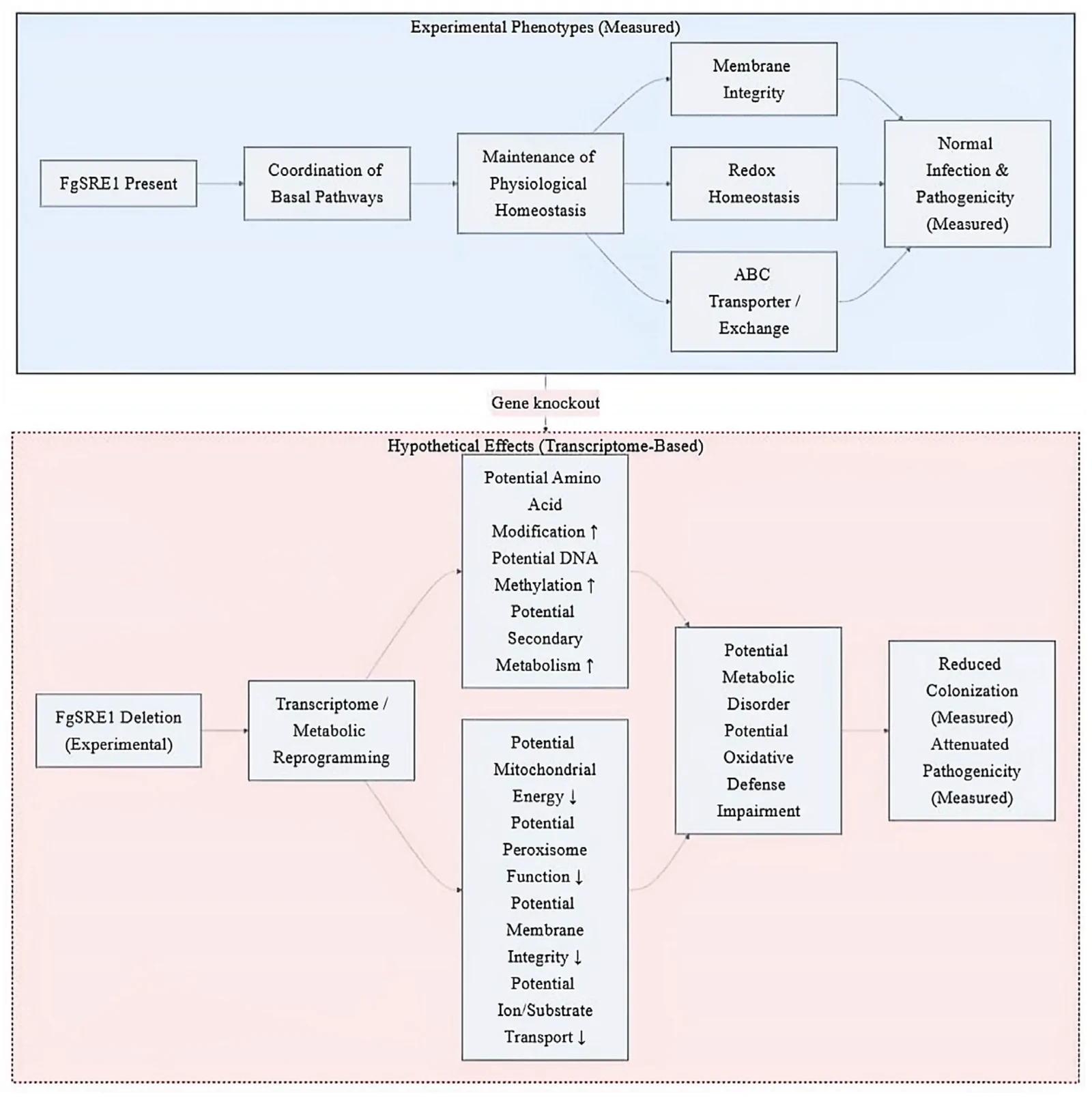
Hypothetical working model based on transcriptomic associations. The model summarizes the proposed roles of *FgSRE1* in *F. graminearum* based on phenotypic observations and transcriptomic data. Upper panel (blue): experimentally measured phenotypes of the wild-type strain (*n* = 3 biological replicates per assay), including normal growth, reproduction, and pathogenicity. Lower panel (red): hypothetical effects inferred from transcriptomic changes observed in the Δ*FgSRE1* mutant (*n* = 3 biological replicates per condition). Solid arrows indicate experimentally confirmed relationships; dashed arrows indicate inferred relationships (transcriptomic changes → potential physiological consequences). Dashed borders in the lower panel denote transcriptome-derived hypotheses. All hypothetical effects—including potential impacts on membrane integrity, redox homeostasis, transport, and secondary metabolism—require direct biochemical validation. The model provides a framework for future experimental validation of the proposed mechanisms.

Based on transcriptomic data and enrichment analyses, we proposed a hypothetical working model for *FgSRE1*-dependent regulation of basal metabolism and pathogenicity in *F. graminearum* (Figure 10). In this model, which is based on transcriptomic correlations rather than experimentally validated mechanisms, we propose that *FgSRE1* may influence lipid and amino acid metabolism, potentially affecting membrane integrity, redox balance, and transmembrane transport, thereby supporting normal infectivity. However, all hypothetical effects—including potential impacts on membrane integrity, oxidative-stress tolerance, and secondary metabolism—require direct biochemical validation. The model is intended as a framework to guide future investigations and does not represent a confirmed regulatory pathway.

*FgSRE1* knockout triggers global transcriptional changes: transferase genes are compensatorily activated, whereas genes associated with energy metabolism, membrane function, and antioxidant defense are repressed. The transcriptomic data suggest that these changes may lead to metabolic disorders and impaired stress defense, potentially hindering fungal colonization and contributing to reduced virulence. However, these inferred relationships require experimental validation.

### 3.10. Quantitative Validation of Differentially Expressed Gene Levels

**Figure 11:**
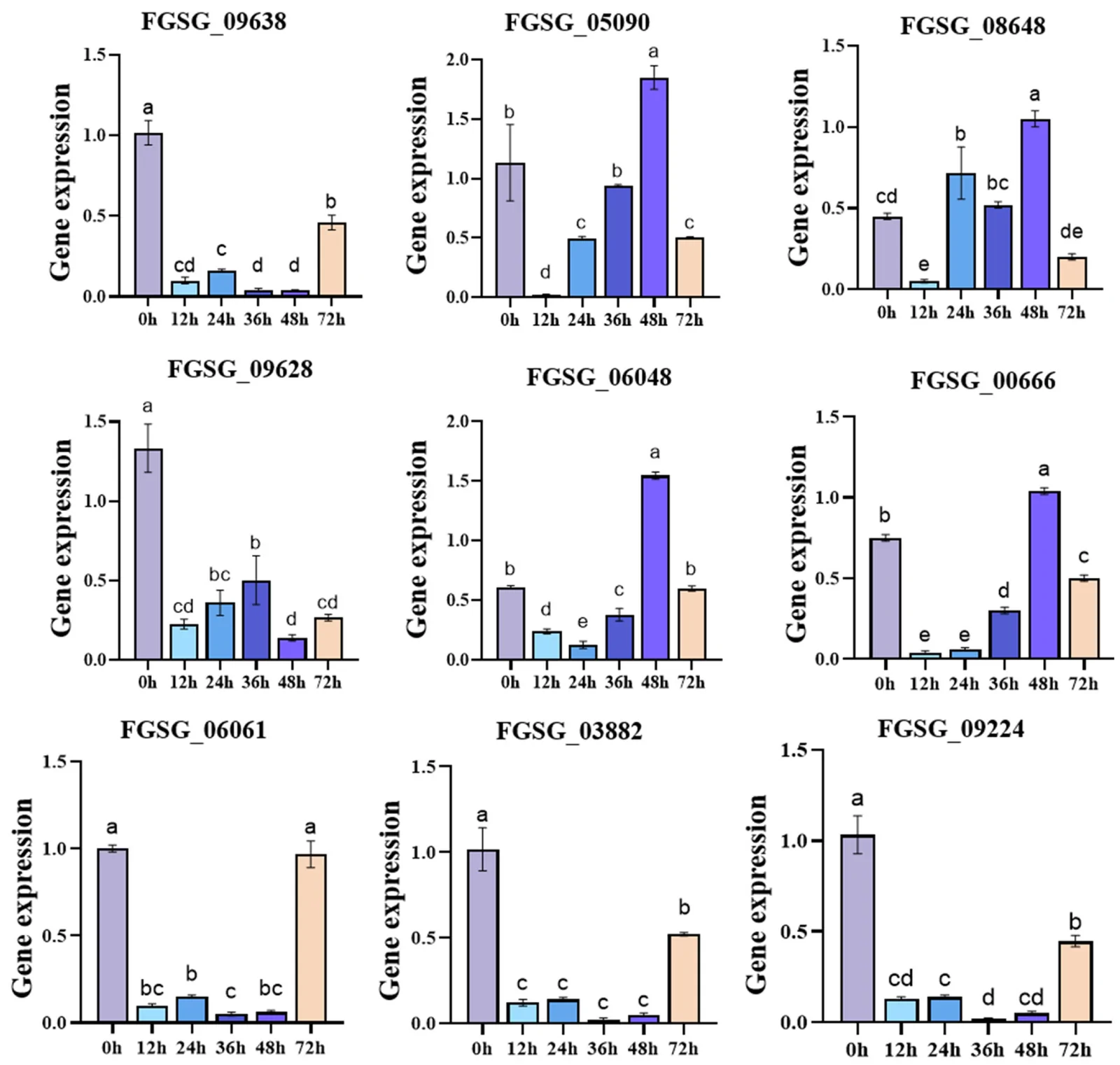
Quantitative verification of differentially expressed genes. qRT-PCR was performed on mycelia collected at 0, 12, 24, 36, 48, and 72 h post-inoculation. The expression profiles of four upregulated genes (FGSG_09638, FGSG_05090, FGSG_08648, and FGSG_09628) and five downregulated genes (FGSG_06048, FGSG_00666, FGSG_06061, FGSG_03882, and FGSG_09224) are shown. Data are presented as mean ± SD (*n* = 3 biological replicates per time point). Data were analyzed using one-way ANOVA followed by Dunnett’s post hoc test, with the 0 h time point as the control. Different letters above the bars indicate statistically significant differences (*p* < 0.05).

To verify the RNA-seq results and to examine the temporal expression patterns of the selected genes, qRT-PCR was performed on mycelia collected at multiple time points (0, 12, 24, 36, 48, and 72 h) post-inoculation. The 48 h time point was selected for detailed comparison with the transcriptomic data, as this was the stage at which FgSRE1 showed its highest expression level during infection (Figure 11). Based on functional categorization and expression patterns identified by RNA-seq, four upregulated genes encoding putative transferases and intracellular molecular modification were selected: FGSG_09638 (encoding putative aspartate carbamoyl transferase), FGSG_05090 (putative leucine carboxyl methyltransferase), FGSG_08648 (putative DNA methyltransferase), and FGSG_09628 (putative diphthine synthase). Five downregulated genes involved in membrane formation, substance transport, and peroxisomal redox homeostasis were also tested: FGSG_06048 (putative mitochondrial ATP synthase subunit), FGSG_00666 (putative peroxisomal membrane protein), FGSG_06061 (putative copper transporter), FGSG_03882 (putative ABC transporter), and FGSG_09224 (putative RTA1-like protein).

All experiments were performed with three independent biological replicates for each sample to guarantee accuracy and repeatability. Data are presented as mean ± standard deviation, and t-tests were applied for statistical analysis. GraphPad Prism 8 was used to generate graphs. The expression profiles from qRT-PCR were consistent with transcriptome data (Figure 11). The opposing expression patterns of the transferase-encoding and membrane/transport-related genes are consistent with the transcriptomic findings, confirming the reliability of the RNA-seq data. However, these qRT-PCR results reflect changes in gene expression levels and do not directly demonstrate membrane dysfunction or impaired transporter activity; direct biochemical assays are required to confirm such functional defects.

## 4. Discussion

*F. graminearum* is a typical soil- and seed-borne phytopathogen that causes destructive diseases in cereal crops, including maize ear rot, which poses persistent risks to grain yield and quality [2,5,31]. Bioinformatic analysis revealed that *FgSRE1* contains a predicted signal peptide and a predicted conserved fungal_RNase domain, suggesting that it is a candidate effector protein of the ribonuclease ribotoxin superfamily. In this study, we demonstrate that *FgSRE1* positively regulates the pathogenicity of *F. graminearum* on maize ears. Deletion of *FgSRE1* significantly reduced spore production, ascospore yields, and virulence on both maize ears and leaves—phenotypes that were fully restored in the complemented strain. These results confirm the functional importance of this effector in *F. graminearum* pathogenesis, consistent with recent reviews summarizing *Fusarium* virulence factors and their roles in fungal pathogenicity [32,33].

Phenotypic assays confirmed that *FgSRE1* deletion impairs both asexual reproduction (conidiation) and sexual reproduction (ascospore formation). However, vegetative growth was unaffected, suggesting that *FgSRE1* specifically regulates developmental processes linked to dissemination and infection rather than general fitness. Recent studies on *F. graminearum* effectors support this functional specificity. For example, the secreted serine carboxypeptidase FgSCP is essential for full virulence, playing critical roles in growth, secondary metabolism, and host invasion [16]. The nucleoside diphosphate kinase FgNdk1 promotes effector secretion and ROS scavenging during infection, and is also required for conidiation and sexual reproduction [34]. Similarly, the effector protease FgTPP1 suppresses immune responses and facilitates disease development by targeting host chloroplasts [15]. Additionally, the nuclear-localized effector FgNls1 is critical for full virulence on wheat [35]. These findings collectively indicate that effectors in *F. graminearum* frequently exhibit dual roles in regulating both developmental processes and virulence, reinforcing the importance of *FgSRE1* as a critical virulence effector.

GO and KEGG enrichment analyses revealed that *FgSRE1* deletion perturbs multiple metabolic pathways under both axenic and infection conditions. Under axenic culture, DEGs were enriched in small-molecule catabolism, proteasome components, membrane constituents, and oxidoreductase activity, with corresponding KEGG pathways including peroxisome, glycerophospholipid metabolism, and amino acid metabolism. Under infection conditions, DEGs were enriched in ion transport, oxidative stress response, mitochondrial membrane components, and ABC transporters, as well as secondary metabolite biosynthesis and tyrosine metabolism.

These transcriptomic changes suggest that *FgSRE1* loss may influence pathways associated with membrane homeostasis and metabolic balance. However, it is important to emphasize that transcriptomic data reveal changes in gene expression, not direct evidence of membrane damage, toxin reduction, or impaired stress tolerance. Direct biochemical assays—such as membrane integrity staining, toxin quantification (e.g., DON and ZEA by HPLC-MS/MS), and oxidative stress tolerance tests—are required to confirm these inferred phenotypes. Therefore, the proposed mechanisms involving membrane disruption, toxin suppression, and oxidative sensitivity remain hypotheses to be tested experimentally.

Our findings align with recent reports on effector functions in *F. graminearum*, supporting the conserved roles of effectors in fungal pathogenesis. Notably, comparative genomic analyses have revealed that *F. graminearum* possesses an expanded open pangenome, and effector content signatures correlate with strain aggressiveness [36]. Additionally, extracellular vesicles have been identified as a mechanism for the unconventional secretion of effectors during corn infection [37]. Genome-wide screens have identified numerous candidate effectors with roles in pathogenicity and virulence [38]. A recent proteomic analysis of the *F. graminearum* secretome in wheat apoplast identified 79 potential secretory proteins, including a cell-death-inducing M43 peptidase [39]. Furthermore, an RGAE homolog in *F. graminearum* is critical for initial infection in wheat and barley [40]. These studies collectively demonstrate the diverse mechanisms by which *F. graminearum* effectors manipulate host plants. In particular, the observation that *FgSRE1* deletion impairs both reproduction and virulence mirrors the dual functions reported for FgSCP and FgNdk1 [16,34], suggesting that candidate effectors in *F. graminearum* often coordinate developmental regulation with pathogenic mechanisms. However, this study provides the first systematic characterization of a predicted RNase-type effector in the context of maize ear rot, extending our understanding of effector diversity beyond the well-studied immune-suppressive effectors.

Several limitations should be acknowledged. First, the subcellular localization of *FgSRE1* within plant cells has not been experimentally verified; whether it localizes to the nucleus, cytoplasm, or specific organelles remains unknown. Second, although *FgSRE1* is predicted to possess RNase activity based on its conserved fungal_RNase domain, enzymatic activity has not been directly measured. Third, while transcriptomic data suggest potential impacts on toxin biosynthesis and membrane integrity, these findings require direct biochemical validation. Fourth, although the complemented strain was verified by GFP fluorescence and phenotypic recovery, molecular confirmation of *FgSRE1* expression restoration by qRT-PCR was not performed in this study. Fifth, all pathogenicity assays were conducted under controlled greenhouse conditions; field experiments are needed to confirm the agronomic relevance of our findings.

Despite these limitations, this study establishes *FgSRE1* as a candidate virulence effector in *F. graminearum* and provides a foundation for future investigations. Functional validation of its RNase activity, identification of its host targets, and elucidation of its secretion mechanism will be important next steps. The transcriptomic data suggest transcriptional perturbation of pathways associated with membrane function and redox homeostasis, but the possible effects on membrane integrity, oxidative-stress tolerance, and mycotoxin production require direct experimental validation. The identification of RNase effectors such as Fg12, which contributes to *F. graminearum* virulence and induces plant cell death through its RNase activity [20], further supports the potential significance of *FgSRE1* as an RNase-type effector. Recent advances in effector identification technologies—including BioID [41] and comparative genomics [37] —provide powerful tools for these future investigations. From an applied perspective, *FgSRE1* represents a promising target for developing novel antifungal strategies and resistant maize germplasm.

## Figures and Tables

**Table 1:** Output statistics of sequencing reads.

Sample	Raw Bases (bp)	Clean Bases (bp)	Effective Rate (%)	Error Rate (%)	Q20 (%)	Q30 (%)	rRNA Ratio (%)
Fg_1	6.31 G	5.94 G	94	0.02	98.11	94.71	0.30
Fg_2	6.02 G	5.62 G	93	0.02	98.37	95.23	0.18
Fg_3	6.32 G	5.94 G	94	0.02	98.19	94.85	0.56
FgHZ_1	6.06 G	5.67 G	94	0.02	98.31	95.21	0.15
FgHZ_2	6.82 G	6.35 G	93	0.02	98.33	95.09	0.10
FgHZ_3	6.52 G	6.08 G	93	0.02	98.21	94.94	0.24
TBRFg_1	6.41 G	6.05 G	94	0.02	98.11	94.66	0.90
TBRFg_2	6.33 G	6.02 G	95	0.02	98.12	94.67	0.18
TBRFg_3	6.24 G	5.72 G	92	0.02	98.15	94.79	0.52
TBRFgHZ_1	7.22 G	5.32 G	74	0.02	98.34	95.42	0.24
TBRFgHZ_2	7.8 G	7.24 G	93	0.02	98.36	95.30	0.15
TBRFgHZ_3	6.15 G	5.73 G	93	0.02	98.45	95.54	0.22

Note: Sample: Sample name; Raw Base (bp): Raw data output, the number of sequencing reads multiplied by the length of each read, in base pairs (bp); Clean Bases (bp): The amount of valid data after filtering, calculated as the number of filtered sequencing reads multiplied by the length of each read, in base pairs (bp); Effective Rate (%): The ratio of clean data to raw data; Error Rate (%): Base error rate; Q20, Q30: The percentage of bases with Phred scores greater than 20 and 30, respectively, out of the total number of bases; rRNA Ratio (%): The proportion of ribosomal RNA (rRNA).

**Table 2:** Statistics on the alignment of samples and reference genomes.

Sample	Total Reads	Total Map	Unique_Map	Multi_Map
Fg_1	39,628,806	33,933,030 (85.63%)	33,753,018 (85.17%)	180,012 (0.45%)
Fg_2	37,476,328	32,643,817 (87.11%)	32,525,183 (86.79%)	118,634 (0.32%)
Fg_3	39,624,476	36,900,184 (93.12%)	36,587,994 (92.34%)	312,190 (0.79%)
FgHZ_1	37,783,120	33,243,166 (87.98%)	33,135,492 (87.7%)	107,674 (0.28%)
FgHZ_2	42,303,740	36,904,753 (87.24%)	36,799,258 (86.99%)	105,495 (0.25%)
FgHZ_3	40,539,854	35,918,066 (88.6%)	35,757,236 (88.2%)	160,830 (0.4%)
TBRFg_1	40,308,184	34,318,104 (85.14%)	34,210,033 (84.87%)	108,071 (0.27%)
TBRFg_2	40,114,580	34,718,256 (86.55%)	34,590,358 (86.23%)	127,898 (0.32%)
TBRFg_3	38,120,688	33,267,134 (87.27%)	33,144,343 (86.95%)	122,791 (0.32%)
TBRFgHZ_1	35,480,956	30,573,937 (86.17%)	30,412,311 (85.71%)	161,626 (0.46%)
TBRFgHZ_2	48,235,400	42,683,723 (88.49%)	42,553,071 (88.22%)	130,652 (0.27%)
TBRFgHZ_3	38,167,682	32,332,701 (84.71%)	32,189,305 (84.34%)	143,396 (0.38%)

Note: sample: Sample name; total_reads: Number of clean reads in the sequencing data after quality control; total_map: Number of reads mapped to the genome and their percentage; unique_map: Number of reads mapped to a single location on the reference genome and their percentage (used for subsequent quantitative data analysis); multi_map: Number of reads mapped to multiple locations on the reference genome and their percentage.

## Data Availability

The RNA-seq data presented in this study have been deposited in the NCBI Sequence Read Archive (SRA) and are available under BioProject accession number PRJNA1511844.

## Supplementary Materials

The following supporting information can be downloaded at https://www.mdpi.com/article/10.3390/jof12080607/s1, Table S1: Primers used for construction of the Δ*FgSRE1* deletion mutant; Figure S1: PCR verification of the Δ*FgSRE1* knockout mutant; Figure S2: Fluorescence localization of *FgSRE1*-GFP in the complemented strain ΔFgSRE1-C; Table S2: Primers used for qRT-PCR in this study.

## References

[1] Ashiq S., Edwards S., Watson A., Back M. (2022). Biofumigation for the Management of *Fusarium graminearum* in a Wheat-Maize Rotation. Pathogens.

[2] Magarini A., Passera A., Ghidoli M., Casati P., Pilu R. (2023). Genetics and Environmental Factors Associated with Resistance to *Fusarium graminearum*, the Causal Agent of Gibberella Ear Rot in Maize. Agronomy.

[3] Sun S., Yu D., Guo M., Tang M., Yan Z., Sun W., Wu A. (2024). The transcription factor FgSfp1 orchestrates mycotoxin deoxynivalenol biosynthesis in *Fusarium graminearum*. Commun. Biol..

[4] Zhang J., Shi H., Yang Y., Zeng C., Jia Z., Ma T., Wu M., Du J., Huang N., Pan G. (2023). Kernel Bioassay Evaluation of Maize Ear Rot and Genome-Wide Association Analysis for Identifying Genetic Loci Associated with Resistance to *Fusarium graminearum* Infection. J. Fungi.

[5] Duan C.X., Cui L., Xia Y., Dong H.Y., Yang Z.H., Hu Q.Y., Sun S., Li X., Zhu Z.D., Wang X.m. (2022). Precise characterization and analysis of maize germplasm resources for resistance to Fusarium ear rot and Gibberella ear rot. Acta Agron. Sin..

[6] Ma Z., Wang J., Wen S., Ren J., Hui H., Huang Y., Yang J., Zhao B., Liu B., Gao Z. (2024). Evaluation of Maize Hybrids for Resistance to Ear Rot Caused by Dominant Fusarium Species in Northeast China. Agronomy.

[7] Gogoi R., Sipani N.S., Rai S.N. (2022). Chemical control of post-flowering stalk rot of maize with new fungicide formulations. Indian Phytopathol..

[8] Mesterhazy A. (2024). Food Safety Aspects of Breeding Maize to Multi-Resistance against the Major (*Fusarium graminearum*, *F. verticillioides*, *Aspergillus flavus*) and Minor Toxigenic Fungi (*Fusarium* spp.) as Well as to Toxin Accumulation, Trends, and Solutions—A Review. J. Fungi.

[9] Wu Y., Xie L., Jiang Y., Li T. (2022). Prediction of effector proteins and their implications in pathogenicity of phytopathogenic filamentous fungi: A review. Int. J. Biol. Macromol..

[10] Gutjahr C. (2025). Outsmarted by fungi. Science.

[11] Le Naour-Vernet M., Lahfa M., Maidment J.H.R., Padilla A., Roumestand C., de Guillen K., Kroj T., Césari S. (2025). Structure-guided insights into the biology of fungal effectors. New Phytol..

[12] Gu X., Cao Z., Li Z., Yu H., Liu W. (2024). Plant immunity suppression by an β-1,3-glucanase of the maize anthracnose pathogen Colletotrichum graminicola. BMC Plant Biol..

[13] Liu M., Wang F., He B., Hu J., Dai Y., Chen W., Yi M., Zhang H., Ye Y., Cui Z. (2024). Targeting Magnaporthe oryzae effector MoErs1 and host papain-like protease OsRD21 interaction to combat rice blast. Nat. Plants.

[14] Yang G., Yang J., Zhang Q., Wang W., Feng L., Zhao L., An B., Wang Q., He C., Luo H. (2022). The effector protein CgNLP1 of Colletotrichum gloeosporioides affects invasion and disrupts nuclear localization of necrosis-induced transcription factor HbMYB8-Like to suppress plant defense signaling. Front. Microbiol..

[15] Darino M., Jaiswal N., Darma R., Kroll E., Urban M., Xiang Y., Srivastava M., Kim H.S., Myers A., Scofield S.R. (2025). The *Fusarium graminearum* effector protease FgTPP1 suppresses immune responses and facilitates Fusarium head blight disease. Mol. Plant-Microbe Interact..

[16] Liu K., Wang X., Qi Y., Li Y., Shi Y., Ren Y., Wang A., Cheng P., Wang B. (2024). Effector Protein Serine Carboxypeptidase FgSCP Is Essential for Full Virulence in *Fusarium graminearum* and Is Involved in Modulating Plant Immune Responses. Phytopathology.

[17] Kalanj N.Ž., Prešern A., Naumoska K., Snoj T., Aupič J., Glavnik V., Albreht A., Mally M., Derganc J., Greimel P. (2025). Plasticity of the cytotoxic Nep1-like protein enables promiscuity in binding to its lipid receptor glycosylinositol phosphorylceramides. Sci. Adv..

[18] Liu T., Song T., Zhang X., Yuan H., Su L., Li W., Xu J., Liu S., Chen L., Chen T. (2014). Unconventionally secreted effectors of two filamentous pathogens target plant salicylate biosynthesis. Nat. Commun..

[19] He S., Huang Y., Sun Y., Liu B., Wang S., Xuan Y., Gao Z. (2022). The secreted ribonuclease SRE1 contributes to Setosphaeria turcica virulence and activates plant immunity. Front. Microbiol..

[20] Yang B., Wang Y., Tian M., Dai K., Zheng W., Liu Z., Yang S., Liu X., Shi D., Zhang H. (2020). Fg12 ribonuclease secretion contributes to *Fusarium graminearum* virulence and induces plant cell death. J. Integr. Plant Biol..

[21] Hao Z., Pan L., Xu J., Yu C., Li J., Luo L. (2025). FGSE02, a Novel Secreted Protein in *Fusarium graminearum* FG-12, Leads to Cell Death in Plant Tissues and Modulates Fungal Virulence. J. Fungi.

[22] Meijueiro M.L., Santoyo F., Ramírez L., Pisabarro A.G. (2014). Transcriptome characteristics of filamentous fungi deduced using high-throughput analytical technologies. Brief. Funct. Genom..

[23] Wang C., Schröder M.S., Hammel S., Butler G., Devaux F. (2016). Using RNA-seq for analysis of differential gene expression in fungal species. Yeast Functional Genomics: Methods and Protocols.

[24] Fan G., Zheng H., Zhang K., Devi Ganeshan V., Opiyo S.O., Liu D., Li M., Li G., Mitchell T.K., Yun Y. (2020). FgHtf1 Regulates Global Gene Expression towards Aerial Mycelium and Conidiophore Formation in the Cereal Fungal Pathogen *Fusarium graminearum*. Appl. Environ. Microbiol..

[25] Kück U., Hoff B. (2010). New tools for the genetic manipulation of filamentous fungi. Appl. Microbiol. Biotechnol..

[26] Ma J., Park S.-W., Kim G., Kim C.S., Chang H.-X., Chilvers M.I., Sang H. (2024). Characterization of SsHog1 and Shk1 Using Efficient Gene Knockout Systems through Repeated Protoplasting and CRISPR/Cas9 Ribonucleoprotein Approaches in Sclerotinia sclerotiorum. J. Agric. Food Chem..

[27] Szabó Z., Pákozdi K., Murvai K., Pusztahelyi T., Kecskeméti Á., Gáspár A., Logrieco A.F., Emri T., Ádám A.L., Leiter É. (2020). FvatfA regulates growth, stress tolerance as well as mycotoxin and pigment productions in Fusarium verticillioides. Appl. Microbiol. Biotechnol..

[28] Chen L., Zhao J., Xia H., Ma Y., Liu Y., Peng M., Xing X., Sun B., Shi Y., Li H. (2020). FpCzf14 is a putative C2H2 transcription factor regulating conidiation in Fusarium pseudograminearum. Phytopathol. Res..

[29] Kim D., Langmead B., Salzberg S.L. (2015). HISAT: A fast spliced aligner with low memory requirements. Nat. Methods.

[30] Love M.I., Huber W., Anders S. (2014). Moderated estimation of fold change and dispersion for RNA-seq data with DESeq2. Genome Biol..

[31] Mourelos C.A., Malbrán I., Mengual Gómez D., Ghiringhelli P.D., Lori G.A. (2024). Dynamics of *Fusarium graminearum* inoculum on residues of naturally infected winter and summer crops. Eur. J. Plant Pathol..

[32] Buttar Z.A., Cheng M., Wei P., Zhang Z., Lv C., Zhu C., Ali N.F., Kang G., Wang D., Zhang K. (2024). Update on the basic understanding of *Fusarium graminearum* virulence factors in common wheat research. Plants.

[33] Gutiérrez-Sánchez A., Plasencia J., Monribot-Villanueva J.L., Rodríguez-Haas B., Ruíz-May E., Guerrero-Analco J.A., Sánchez-Rangel D. (2023). Virulence factors of the genus Fusarium with targets in plants. Microbiol. Res..

[34] Sun M., Zhang Y., Yang Y., Xie X., Zheng H., Dai P., Cao Z., Dong J. (2025). FgNdk1 Promotes Effector Secretion to Scavenge ROS During *Fusarium graminearum* Infection. Mol. Plant Pathol..

[35] Hao G., Naumann T.A., Chen H., Bai G., McCormick S., Kim H.-S., Tian B., Trick H.N., Naldrett M.J., Proctor R. (2023). *Fusarium graminearum* Effector FgNls1 Targets Plant Nuclei to Induce Wheat Head Blight. Mol. Plant-Microbe Interact..

[36] Alouane T., Rimbert H., Bormann J., González-Montiel G.A., Loesgen S., Schäfer W., Freitag M., Langin T., Bonhomme L. (2021). Comparative Genomics of Eight *Fusarium graminearum* Strains with Contrasting Aggressiveness Reveals an Expanded Open Pangenome and Extended Effector Content Signatures. Int. J. Mol. Sci..

[37] Garcia-Ceron D., Lowe R.G.T., McKenna J.A., Brain L.M., Dawson C.S., Clark B., Berkowitz O., Faou P., Whelan J., Bleackley M.R. (2021). Extracellular Vesicles from *Fusarium graminearum* Contain Protein Effectors Expressed during Infection of Corn. J. Fungi.

[38] Fatima M., Anjum Bhat H., Rebekah N., Murugasamy S., Makandar R. (2024). Genome-wide search and gene expression studies reveal candidate effectors with a role in pathogenicity and virulence in *Fusarium graminearum*. Mycologia.

[39] Li P., Zhao R., Fang Y., Fan Y., Hu Q., Huang W., Ma W., Zhang C. (2025). Proteomic Analysis of the *Fusarium graminearum* Secretory Proteins in Wheat Apoplast Reveals a Cell-Death-Inducing M43 Peptidase. J. Fungi.

[40] Rhoades N., Naumann T.A., Kim H.-S., Yulfo-Soto G., McCormick S., Bowman M.J., Vaughan M., Hao G. (2025). An RGAE homolog in *Fusarium graminearum* is critical for initial infection in wheat and barley. Mol. Plant-Microbe Interact..

[41] Miltenburg M.G., Bonner C., Hepworth S., Huang M., Rampitsch C., Subramaniam R. (2022). Proximity-dependent biotinylation identifies a suite of candidate effector proteins from *Fusarium graminearum*. Plant J..

