# Supplementary material for: Deletion of FgSRE1 Impairs Reproduction and Virulence of Fusarium graminearum and Alters Metabolic and Membrane-Transport Gene Expression
Source: J Fungi (Basel). 2026 Aug 14;12(8):607. doi: 10.3390/jof12080607 (PMC13514134; doi:10.3390/jof12080607)
Supplement: Supplementary file 1 [file jof-12-00607-s001.zip › jof-4428341-supplementary.pdf]

Journal of Fungi

Table S1. Primers used for construction of the Δ*FgSRE1* deletion mutant.

primer names	Sequence (5′→3′)
ITS4	TCCTCCGCTTATTGATATGC
V9G	TTACGTCCCTGCCCTTTGTA
FG1F	CGTTGCTTCTAAGATTGCAG
FG2R	CGGTGAGTTCAGGCTTTTTCATCTTGTGAAACGGTCTGTTGA
FG3F	AATAGAGTAGATGCCGACCGGATCACCCTCACTGCAATTAC
FG4R	TATGTAAGTTGCACGACCAC
FG5F	CGGAATTCCATGCTCTTCTTCAAGTCTATCG
FG6R	CCAAGCTTTTAGCTAGTGCCAGTGCAG
hph-f	CCGGTCGGCATCTACTCTATT
hph-r	ATGAAAAAGCCTGAACTCACCG
Yg-r	CATTGTTGGAGCCGAAATCC
Hy-f	CGCAAGGAATCGGTCAATAC
Hph700-f	GACCTGCCTGAAACCGAACT
Hph700-r	GCGTCTGCTGCTCCATACAA
FG7F	CAGGCAAGGCTATTGTGG
RByg-r	ATCCGGTCGGCATCTACT
LBhy-f	CGGAAGTGCTTGACATTG
FG8R	TTCTTTACTCAGGTCCCATT
HBFg-F	AATCATTTGGAGGGCATCA
HBFg-R	CTATTTACAAGAAACGCATCG
YZHBFg-F	TCGAGGGGGGGCCCGGTACCCTTTCCCTCCTTAGTCGC
YZHBFg-R	CCCTTGCTCACCATGGTACCGCTAGTGCCAGTGCAGCC
HB-PLUE-f1	CCTCGAGGGGGGGCCCGGTACCCATGGAGCCGATAAGAGG
HB-PLUE-r1	TCGCCCTTGCTCACCATGGTACCGCTAGTGCCAGTGCAGCC A
HB-PLUE-f2	CGCGGATCCTCAATGGCCTCGCGCAA
HB-PLUE-r2	CGGGGTACCGCTAGTGCCAGTGCAGCCAA
YZ-Plue-R	GTCAGCTTGCCGTAGGTGGCA
YZ-Plue-F	CTGCAGGAATTCGATATCAAGC
Tublin-f	GGTCAGTGCGGTAACCAAATCG
Tublin-r	GGAGCTCAGAGGTGCCGTTGTAA
RtFG-F	CTCTTCTTCAAGTCTATCGTCT
RtFG-R	CCTGAGCAGCAGTGTAGC

https://doi.org/10.3390/jof12080607

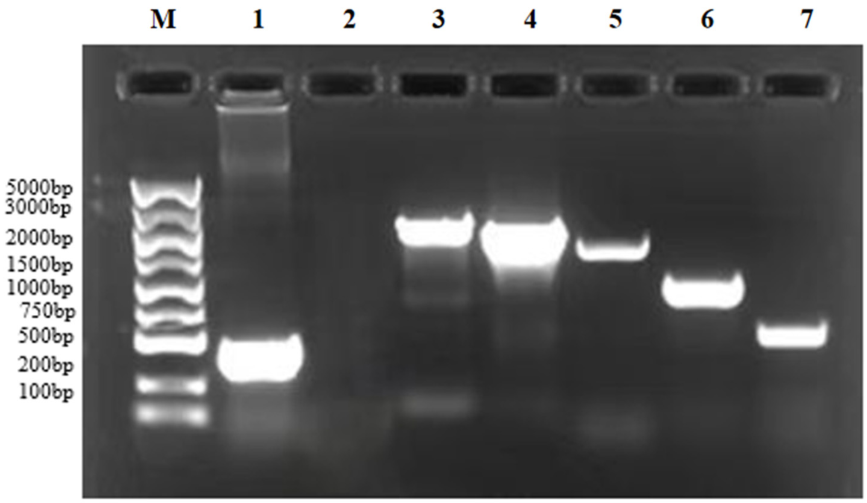

Figure S1. PCR verification of the Δ*FgSRE1* knockout mutant. M: DNA marker; lane 1: hygromycin partial fragment (436 bp); lane 2: target gene detection (no band, confirming gene deletion); lane 3: upstream recombination fragment (2591 bp); lane 4: downstream recombination fragment (2115 bp); lane 5: hygromycin recombination fragment; lane 6: hygromycin fragment (953 bp); lane 7: wild-type (WT) control (target gene band at 527 bp). The absence of the target gene band in lane 2 and its presence in the WT control (lane 7) confirm the successful replacement of *FgSRE1* by the hygromycin resistance cassette. The correct sizes of all amplified fragments verify the successful construction of the Δ*FgSRE1* knockout mutant.

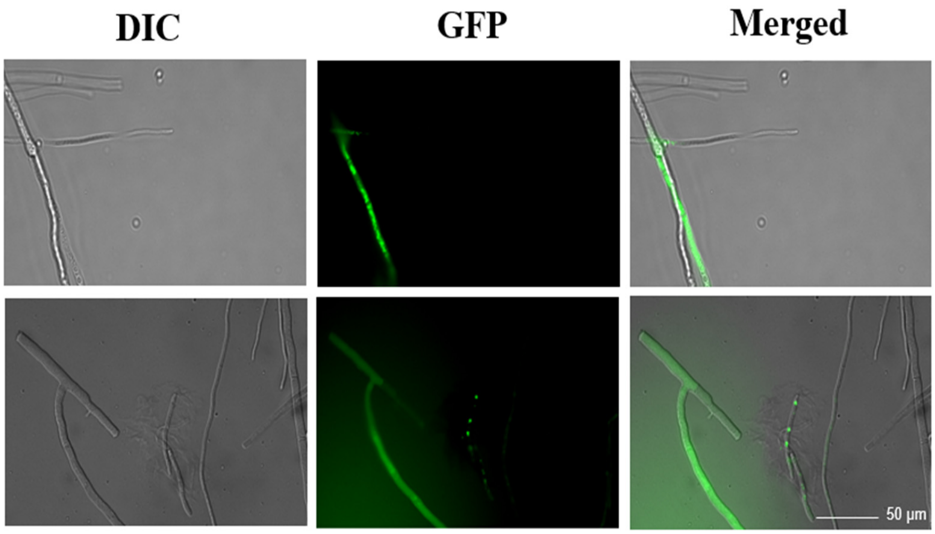

Figure S2 Fluorescence localization of FgSRE1-GFP in the complemented strain ΔFgSRE1-C.DIC, differential interference contrast channel; GFP, GFP fluorescence channel; Merged, the overlay of DIC and GFP signals. Scale bar = 50 μm.

Table S2. Primers used for qRT-PCR in this study.

primer names	Sequence (5′→3′)
MFS1F	GAGCAGGTCGAATGGGTC
MFS1R	CGATTGAGGTGGCTTGTAG
MFS2F	TATCTGGATGACCCTTATG
MFS2R	GTCTGCACTGAGGAATGA
MFS3F	TCGTGGACATTAACCTCT
MFS3R	AACTTCGTCAGACACCTT
MFS4F	TCGTTGGTGATCCTTTTG
MFS4R	GGACATGATGGAGGCATT
MFX1F	AAGGCCGCTCGCCCTGCTGT
MFX1R	GGAGAAGAGTCTGACGCTTCATG
MFX2F	AGCGTTCTGATTGACGGTAG
MFX2R	CCTAAGCCCTGGGAGCAA
MFX3F	GGACCACGGAGCTATGGG
MFX3R	CAAGGAAGCAGGCATCAA
MFX4F	TTGAGCGGTATCAGGGAA
MFX4R	CCGAGGCTGATGATGTATG
MFX5F	ATTGTTGCGGCTGTGATA
MFX5R	AAGGGAAGACTGTCTGGTTG

https://doi.org/10.3390/jof12080607
